# Modeling the Energy Landscape of Side Reactions in the Cytochrome bc_1_ Complex

**DOI:** 10.3389/fchem.2021.643796

**Published:** 2021-05-19

**Authors:** Peter Husen, Ilia A. Solov’yov

**Affiliations:** ^1^ Department of Physics, Chemistry and Pharmacy, University of Southern Denmark, Odense, Denmark; ^2^ Department of Physics, Carl von Ossietzky Universität Oldenburg, Oldenburg, Germany

**Keywords:** electron transfer, computational biophysics, molecular dynamics, superoxide, free energy perturbation, quantum chemical modeling, enzymes, proteins

## Abstract

Much of the metabolic molecular machinery responsible for energy transduction processes in living organisms revolves around a series of electron and proton transfer processes. The highly redox active enzymes can, however, also pose a risk of unwanted side reactions leading to reactive oxygen species, which are harmful to cells and are a factor in aging and age-related diseases. Using extensive quantum and classical computational modeling, we here show evidence of a particular superoxide production mechanism through stray reactions between molecular oxygen and a semiquinone reaction intermediate bound in the mitochondrial complex III of the electron transport chain, also known as the cytochrome $bc_{1}$ complex. Free energy calculations indicate a favorable electron transfer from semiquinone occurring at low rates under normal circumstances. Furthermore, simulations of the product state reveal that superoxide formed at the Q_*o*_-site exclusively leaves the $bc_{1}$ complex at the positive side of the membrane and escapes into the intermembrane space of mitochondria, providing a critical clue in further studies of the harmful effects of mitochondrial superoxide production.

## 1 Introduction

**FIGURE 1:**
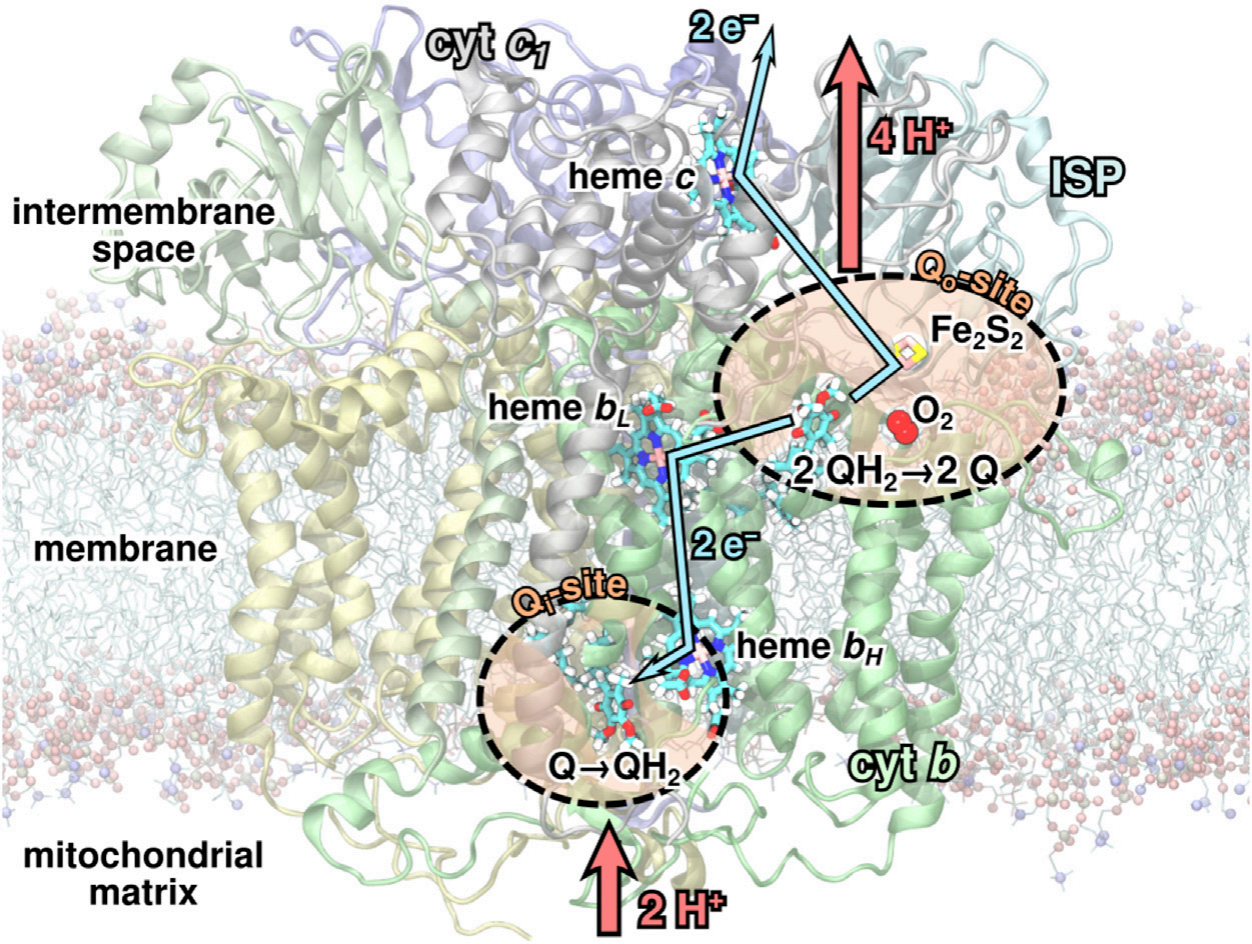
The $bc_{1}$ complex and its reaction cycle. During the Q-cycle, two substrate quinol molecules (QH_2_) are oxidized to quinone (Q) at the Q_*o*_-site, while one Q is reduced to QH_2_ at the Q_*i*_-site of the $bc_{1}$ complex. In this process, two protons are absorbed from the negative side of the membrane, and four are released to the positive side, hence maintaining the transmembrane electrochemical gradient. An oxygen molecule may occasionally bind in a pocket near the Q_*o*_-site (Husen and Solov’yov, 2016), which could lead to superoxide production.

The cytochrome $bc_{1}$ complex is a transmembrane protein complex in the inner mitochondrial membrane of eukaryotes or the plasma membrane of photosynthetically active bacteria. Through its sophisticated reaction cycle, the Q-cycle (Mitchell, 1975; Lhee et al., 2010; Crofts et al., 2017; Barragan et al., 2021) depicted in Figure 1, it serves as a crucial energy transducer in the ATP synthesis pathway. It is, however, also suspected to be a source of harmful superoxide production through stray reactions with molecular oxygen, O_2_ (Cape et al., 2007; Dröse and Brandt, 2008; Rottenberg et al., 2009; Brand, 2010; Guillaud et al., 2014).

As part of the Q-cycle, an ubiquinol (QH_2_) molecule from the membrane is oxidized to ubiquinone (Q) in a bifurcated reaction at one of the two substrate binding sites, the Q_*o*_-site, of the $bc_{1}$ complex. It was previously shown (Husen and Solov’yov, 2016, Husen and Solov’yov, 2017) that O_2_ molecules can occasionally enter the protein complex through the membrane and become trapped close to the iron-sulfur cluster (Fe_2_S_2_) at the Q_*o*_ binding site of the $bc_{1}$ complex during a short-lived state of the Q-cycle, in which a bound QH_2_ has been partly oxidized to a semiquinone anion (${\text{Q}}^{•-}$) at the Q_*o*_-site (Crofts et al., 2003; Barragan et al., 2016). As this reaction intermediate is a radical, it is conceivable that it could react with a nearby oxygen molecule to produce a potentially harmful superoxide anion, $O_{2}^{•-}$.

Earlier computational studies (Salo et al., 2017) have indicated two candidate mechanisms of superoxide production in the $bc_{1}$ complex initiated from a state with a radical semiquinone anion bound at the Q_*o*_-site of the complex and an oxygen molecule trapped at the previously identified binding pocket near Fe_2_S_2_ Husen and Solov’yov (2016). In the present investigation, these are studied separately as *model I*, in which an electron is transferred from semiquinone to the O_2_ molecule,

$${\text{Q}}^{•-}+{\text{O}}_{2}\to \text{Q}+{\text{O}}_{2}^{•-},$$ (1)

and *model II*, in which the electron is instead transferred from the iron-sulfur cluster (Fe_2_S_2_) in the iron-sulfur protein (ISP) subunit of the $bc_{1}$ complex:

$${\text{Fe}}_{2}{\text{S}}_{2}^{-}+{\text{O}}_{2}\to {\text{Fe}}_{2}{\text{S}}_{2}+{\text{O}}_{2}^{•-}.$$ (2)

**FIGURE 2:**
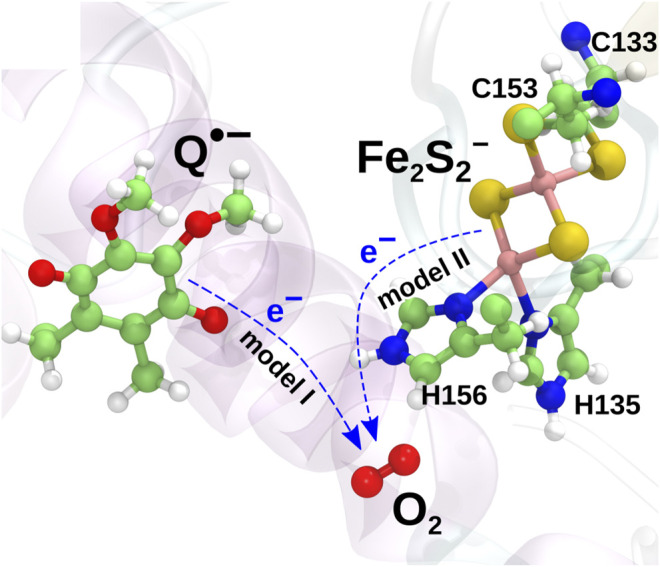
Two charge transfer mechanisms possibly leading to superoxide production. In *model I*, an electron is transferred from the radical semiquinone anion to the O_2_ molecule, oxidizing the semiquinone to a neutral quinone. In *model II*, an electron is instead transferred from Fe_2_S_2_^−^ to O_2_ to yield a superoxide radical.

The two models are illustrated in Figure 2. The mechanism described in model II would appear to be prohibited due to the high midpoint redox potential of Fe_2_S_2_ and can essentially be ruled out from the experimental evidence (Sun and Trumpower, 2003; Bleier and Dröse, 2013). It has, however, been included in the present investigation for completeness, as the reaction is consistently observed in both earlier quantum chemical calculations (Salo et al., 2017) and new extended calculations presented here. The inclusion of this implausible reaction will serve as a test for the model and to shed light on the importance of the local environment, when modeling electron transfer processes.

Studying complex biomolecular processes computationally generally require a range of computational modeling tools covering different time and length scales (Korol et al., 2020). The present work employs several computational modeling techniques at both the quantum and classical level to characterize the possible reactions leading to superoxide formation at the Q_*o*_-site of the $bc_{1}$ complex. Essentially, this study aims to complete an endeavor to computationally model superoxide production at the Q_*o*_-site of the $bc_{1}$ complex from O_2_ binding to $O_{2}^{•-}$ unbinding, specifically with anionic semiquinone at the Q_*o*_-site (Husen and Solov’yov, 2016, Husen and Solov’yov, 2017; Salo et al., 2017) to gauge the importance of this mechanism as a contributor to metabolic superoxide production. Firstly, previously reported (Salo et al., 2017) quantum chemical (QC) calculations are repeated with significantly extended sampling over an ensemble of molecular dynamics (MD) simulation snapshots from a previous study (Husen and Solov’yov, 2016) with O_2_ bound near Fe_2_S_2_, leading to a much more clear classification of electron transfer reactions at the Q_*o*_-site into the two main reactions, models I and II described above, with superoxide as a product. The same calculations are also repeated with a modified cluster model to gauge the sensitivity to the local protein environment of the QC calculations. Secondly, extended MD simulation of the product state of the modeled reactions are carried out to study the binding time and unbinding of superoxide. Lastly, the free energy landscapes of the two reactions are studied using two separate MD-based approaches in order to gauge the feasibility of the reactions and estimate possible rates.

The protonation state of semiquinone at the Q_*o*_-site is debated (Pietras et al., 2016), and the choice of an anionic semiquinone in our model is based on the assumption that it would be the most reactive with oxygen, and some experimental indications exist of this form of semiquinone (Mulkidjanian, 2005; Cape et al., 2007). The anionic form is also interesting for superoxide production, as a proposed protective complex (Pietras et al., 2016; Bujnowicz et al., 2019) with a hydrogen bond between ${\text{QH}}^{•}$ and the ISP could not be formed in this state, so if both are possible during the Q-cycle, the anionic form would likely be the most relevant for superoxide production. Similarly, the semiquinone at the Q_*o*_-site could alternatively be a result of a semireverse mechanism, where QH_2_ has rapidly donated its second electron to heme b_*L*_ in the cytochrome b subunit, in accordance with the Q-cycle, but the electron is then later transferred back to the oxidized quinone (Dröse and Brandt, 2008; Sarewicz et al., 2010). The semireverse reaction is in principle also consistent with our model, but it places fewer constraints on the state and position of the Fe_2_S_2_-cluster, as it can happen with more delay. Separate computational investigations using the same methodology would be warranted to study mechanisms involving the semireverse reaction or a neutral semiquinone at the Q_*o*_-site as possible contributors to superoxide production.

The combined investigations include all levels of modeling of an important biomolecular process: Diffusion of O_2_, the quantum chemistry of electron transfer and the free energy budget of the complex protein-ligand enviroment of the hypothetical reaction. Experimental methods can only address the problem much more indirectly, so the details we can get out of modeling are essential to make sure we understand the mechanism and for this, the completeness of the model is essential to allow comparison with experiment.

## 2 Computational Methods

The computational protocols for the quantum chemical modeling and all-atom MD simulations employed in the present work are described in the following sections.

### 2.1 Quantum Chemical Calculations

**FIGURE 3:**
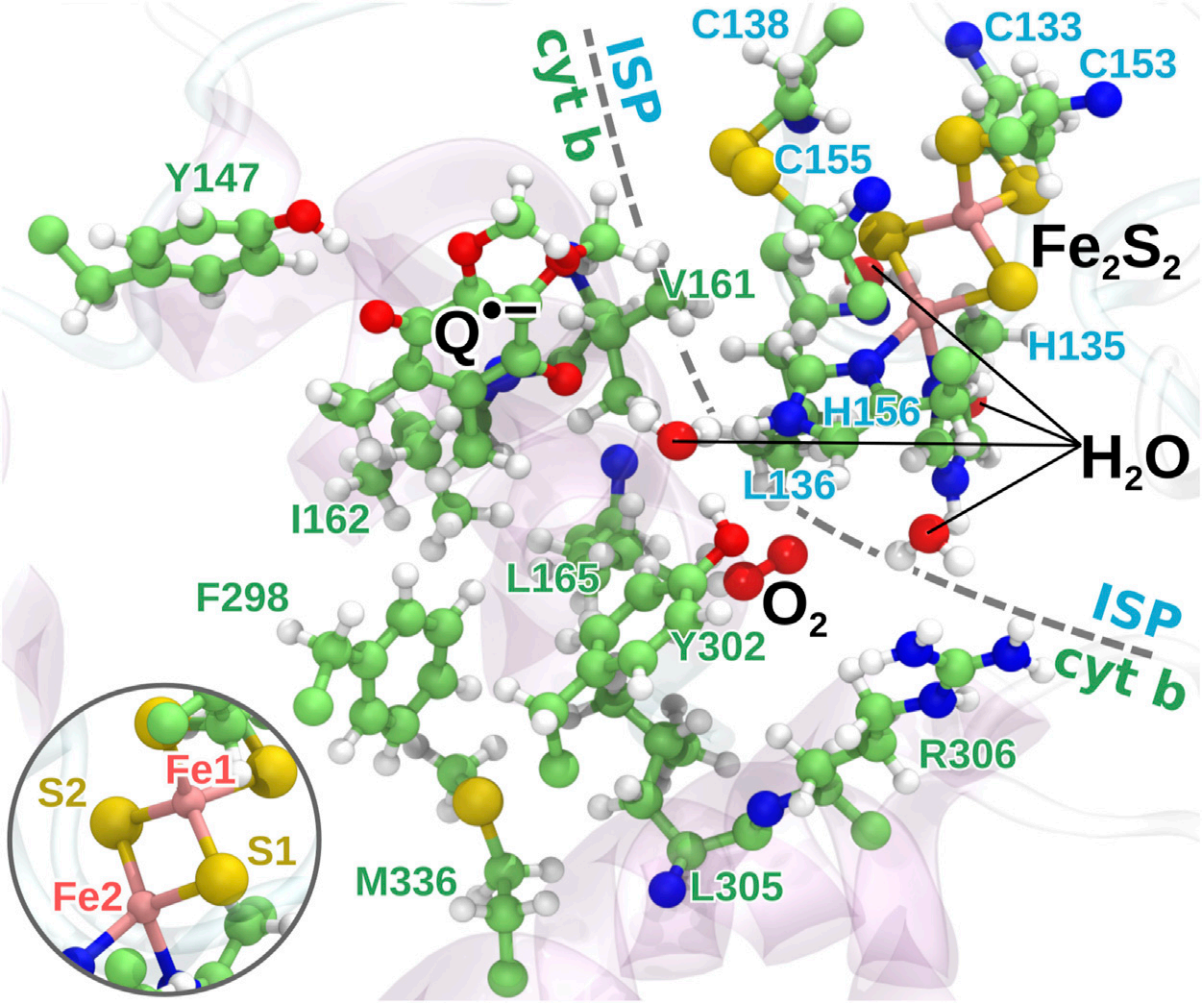
The quantum region used for QC calculations. The region consists of the head group of the semiquinone anion, the iron-sulfur cluster, 16 amino acid residues from the protein, the bound O_2_ molecule and four water molecules. The two iron atoms are modeled as having four and five unpaired electrons, respectively, with anti-parallel spin orientation between the two atoms as an initial guess for the QC calculations. Inset: the naming of the iron and sulfur atoms of Fe_2_S_2_ used in the model.

The quantum chemical calculations followed the protocol of an earlier study (Salo et al., 2017), but with a significantly increased number of included snapshots to allow improved statistics following the aim of the present investigation. A total of 200 snapshots from an earlier MD simulation (Husen and Solov’yov, 2016) of the membrane embedded $bc_{1}$ complex from *Rhodobacter capsulatus* (PDB ID: 1ZRT (Berry et al., 2004)) in presence of molecular oxygen were extracted at random among the parts of the simulated trajectory with O_2_ present in the binding pocket near Fe_2_S_2_ (see Supplementary Figure S5 in the supplementary material (SM). A region, illustrated in Figure 3, including the bound semiquinone and oxygen molecule, the iron-sulfur cluster and a number of nearby amino acids was extracted from each snapshots and used for quantum chemical calculations. Water molecules were also observed in the vicinity of the O_2_ binding position in the earlier simulations (Husen and Solov’yov, 2016), so for each extracted snapshot, the four water molecules closest to the midpoint between TYR302 of the cytochrome b subunit and HIS135 of the ISP subunit were also included in the studied quantum region. The unsaturated bonds in the amino acids were capped with hydrogen. A separate set of calculations was also carried out with the quantum region extended to included the residues V293, P294 and E295 of the cythocrome b subunit. These results of the extended calculations are presented in the SM.

Quantum chemical geometry optimization calculations were carried out in Gaussian 09 (Frisch et al., 2013) using the CAM-B3LYP density functional theory method (Yanai et al., 2004) with the 6-31G basis set (Rassolov et al., 2001) on the extracted quantum region from each sampled MD snapshot. Backbone carbons of the amino acids as well as the carbon atom connecting the ring and the tail of $O_{2}^{•-}$ were held fixed during the optimization in order to impose the overall conformation sampled from MD, while still allowing the system to relax according to the quantum model. The quantum region was divided into fragments to allow setting the charge and multiplicity for the different molecules and amino acids separately in the initial wavefunction guess. Furthermore, the iron-sulfur cluster was split into separate fragments for each of the four atoms to allow modeling the anti-ferromagnetic coupling between the two iron atoms (Szilagyi and Winslow, 2006). The multiplicities for the two iron atoms were set to five and six, respectively, with anti-parallel spins between the two. The multiplicity of five for one of the irons was due to the fact that this iron atom has accepted the first electron from the bound QH_2_.

The relatively simple 6-31G basis set with no polarization or diffuse functions was chosen despite the inclusion of iron atoms in the model due to the size of the quantum region with a total of 263 atoms, which would have made the calculations unrealistic with a more advanced basis set. The 6-31G basis set has, however, been shown to be able to model the anti-ferromagnetic coupling in the Fe_2_S_2_-cluster (Szilagyi and Winslow, 2006) and the pKa of its coordinating histidine residues of the ISP (Barragan et al., 2015) correctly, indicating that a reasonable quantum model of the Fe_2_S_2_-cluster and its surroundings can be achieved using the basis set.

### 2.2 Molecular Dynamics Simulations

The MD simulation protocol from earlier studies (Barragan et al., 2015; Husen and Solov’yov, 2016) was adopted, such that equilibrated structures and simulated trajectories from those studies were employed for the further modeling and analysis in the present investigation. Briefly, the X-ray crystal structure of the $bc_{1}$ complex from *Rhodobacter capsulatus* (PDB ID: 1ZRT (Berry et al., 2004)) was embedded in a membrane patch consisting of a mixture of phosphatidylcholine (PC 18:2/18:2), phosphatidylethanolamine (PE 18:2/18:2) and cardiolipin (CL 18:2/18:2/18:2) with a total of 102 CL, 406 PC and 342 PE lipids. The histidines of the protein were all considered δ-protonated, except for H135 and H156 of the ISP, which are coordinating the Fe_2_S_2_-cluster through the N_*δ*_-atom. These are instead modeled as ϵ-protonated. Two disulfide bonds were introduced based on inspection of the crystal structure: between C144 and C167 of the cytochrome c_1_ subunit and between C138 and C155 of the ISP. The protonation state of H156 upon quinol binding is debated (Crofts et al., 1999; Postila et al., 2013), but as we are modeling the semiquinone state, the histidine would be protonated in either case. Similarly, E295 of cytochrome b was considered as protonated as it is expected to have received the second proton from oxidation of QH_2_. Finally, ASP252 of cytochrome b was considered protonated as it appears to form a hydrogen bond with PHE248 in the crystal structure. Both protonation states of ASP252 could be relevant in a proposed switching mechanism (Postila et al., 2016) involved in proton transport at the Q_*i*_-site.

The system was solvated in a water box of 197 Å×177 Å×142 Å size, and a concentration of 0.05 mol/L NaCl was added, resulting in a total of 497,562 atoms. In the adopted equilibrated structures (Barragan et al., 2015; Husen and Solov’yov, 2016), the membrane embedded $bc_{1}$ complex exhibits a stable conformation, where the transmembrane helical scaffold is filled by the Q_*i*_-site quinones and lipids from the membrane. No water molecules are observed in the transmembrane region. While interactions between cardiolipin and the Q_*i*_-site of the $bc_{1}$ complex are reported in the literature (Catucci et al., 2012; Postila et al., 2016), no cardiolipin was found near the Q_*i*_-site in the equilibrated structure. This is most likely due to the fact that lipids where randomly placed in the original simulations (Barragan et al., 2015; Husen and Solov’yov, 2016), and the timescale of lipid diffusion in the membrane is too great to observe proper mixing in realistic MD simulations. However, as the present study focuses on the Q_*o*_-site activity, we are mostly concerned with the overall structural stability in the transmembrane region.

In the present study, the $bc_{1}$ complex was modeled using MD simulations in three different redox states: The reactant state with semiquinone at the Q_*o*_-site and O_2_ bound and the product state in model I and II, respectively, with a newly formed superoxide bound. The position of the bound O_2_ or $O_{2}^{•-}$ molecule is depicted in Supplementary Figure S5 in the SM. All earlier and new simulations have quinone at the Q_*i*_-site. The simulated trajectories for the reactant state were taken from an earlier study (Husen and Solov’yov, 2016), while the simulations of the product state in the two models were carried out in the present investigation. The overall simulation protocol including both previous and new simulations is shown in Supplementary Table S1.

The reactant state simulations (Husen and Solov’yov, 2016) included a concentration of molecular oxygen added initially to the water phase in the simulation box to study the dynamics O_2_ and identify its potential binding sites in the $bc_{1}$ complex. The simulated trajectories were analyzed to identify events of O_2_ binding at a particular site near ${\text{Q}}^{•-}$ and Fe_2_S_2_ at the Q_*o*_-site (see Supplementary Figure S5), and the parts of the trajectories with a bound O_2_ molecule were extracted and used for the energy sampling in the present investigation and for setting up quantum chemical calculations and product state simulations. A binding event was identified as starting when an oxygen molecule comes within a distance of 9 Å from the Fe_2_S_2_-cluster and ending when it is last observed within 13 Å of Fe_2_S_2_ before reaching a threshold distance of 20 Å. This definition allows O_2_ to temporarily fluctuate more than 13 Å away, as long as it returns again rather than leaving entirely.

The product state simulations were set up by choosing a simulation snapshot with a trapped O_2_ molecule in the binding pocket near Fe_2_S_2_ and modifying the atomic partial charges and force field parameters to model a product state according to model I and II, respectively, based on earlier parametrizations (Kaszuba et al., 2013; Husen and Solov’yov, 2016) of the involved molecular constituents in different redox states. In model I, the Q_*o*_-site was modeled with a neutral quinone and a negatively charged iron-sulfur cluster, while in model II, a semiquinone anion and a neutral Fe_2_S_2_ was assumed, as specified in Supplementary Table S1. In both cases, the bound O_2_ was changed to $O_{2}^{•-}$. For each model, 100 replicate MD simulations with $O_{2}^{•-}$ initially bound were then carried out for long enough to observe it unbind, following an approach previously used to model O_2_ binding in proteins (Husen et al., 2019).

MD simulations were performed using NAMD (Phillips et al., 2005) with the CHARMM 36 (MacKerell et al., 1998, MacKerell et al., 2004; Best et al., 2012) force field, and VMD (Humphrey et al., 1996) was used extensively for system construction, data analysis and visualization. The force field parameters and partial charges for the heme groups, iron-sulfur clusters and bound quinones and semiquinones were taken from an earlier study (Kaszuba et al., 2013; Postila et al., 2013), except for the partial charges of Fe_2_S_2_^−^ and ${\text{Q}}^{•-}$ which were obtained separately (Husen and Solov’yov, 2016).

### 2.3 Free Energy Perturbation Simulations

The free energy perturbation (FEP) simulations (Dixit and Chipot, 2001; Woo and Roux, 2005) follow the same protocol as the MD simulations described above, except that constant pressure, rather than constant volume, is employed in the simulations, and electrostatic interactions were modulated using the alchemical transformation method in NAMD. FEP transformations, where electrostatic interactions were gradually turned on or off through a coupling parameter, λ, were performed to measure the free energy change due to going from reactant to product state, when modeling the reactions in Eqs. 1, 2 for model I and II, respectively. The van der Waals and bonded interactions were assumed to be essentially unchanged after electron transfer, so the same force-field parameters were used for the product and reactant states, and the van der Waals and bonded interactions were kept fully coupled during the transformations, which simplifies the method considerably. The FEP transformations were carried out in two parts – first turning off electrostatic interactions in the reactant configuration with O_2_ bound at the binding pocket near Fe_2_S_2_, FEP1, and then turning the interactions back on in the product configuration with $O_{2}^{•-}$ bound in place of O_2_ FEP2:

$${\text{Q}}^{•-}+{\text{O}}_{2}\overset{\text{FEP}1}{\overset{\text{forward}}{\underset{\text{backward}}{⇌}}}\;{\text{Q}}^{\left(0\right)}+{\text{O}}_{2}\;\overset{\text{FEP}2}{\overset{\text{forward}}{\underset{\text{backward}}{⇌}}}\;\text{Q}+{\text{O}}_{2}^{•-}\;\left(\text{model I}\right),$$ (3)

$${\text{Fe}}_{2}{\text{S}}_{2}^{-}+{\text{O}}_{2}\overset{\text{FEP}1}{\overset{\text{forward}}{\underset{\text{backward}}{⇌}}}\;{\text{Fe}}_{2}{\text{S}}_{2}^{\left(0\right)}+{\text{O}}_{2}\overset{\text{FEP}2}{\overset{\text{forward}}{\underset{\text{backward}}{⇌}}}{\text{Fe}}_{2}{\text{S}}_{2}+{\text{O}}_{2}^{•-}\;\left(\text{model II}\right),$$ (4)

where the “(0)” superscript indicates that all electrostatic interactions are turned off, equivalent to setting all partial charges of atoms to zero.

For model I, the part of the system undergoing the FEP transformation included ${\text{Q}}^{•-}$ at the Q_*o*_-site and the nearby bound O_2_ molecule, while for model II, the FEP region included O_2_, the iron-sulfur cluster and its coordinating cysteine and histidine residues as well as SER158 of the ISP (implicitly included with Fe_2_S_2_ in Eq. 2), as the partial charges on these residues differ between the modeled charge states of the Fe_2_S_2_ cluster. Each FEP transformation was carried out in both forward and backward mode, i.e. stepping the coupling parameter, λ, from 0 to 1 and then back again to 0 in 16 steps for each transformation. The simulations were carried out with three settings of the simulation time per λ-window, 10 ps, 1 and 2 ns, to test the sensitivity of the results to the simulation length. The resulting set of 24 FEP calculations is illustrated in Supplementary Figure S1: two model reactions, each modeled through two partial transformations (FEP1 and FEP2) and all transformations carried out in both forward and backward mode and for three choices of simulation length. Backward transformations were carried out starting from the atomic coordinates resulting from the corresponding forward transformations, and the FEP2 forward transformations were continued from the end of the FEP1 forward transformations as also illustrated in the figure. The O_2_ molecule was restrained to move within a sphere of radius 8 Å and 10 Å for model I and II, respectively, to prevent it from leaving the binding site. For model I, the center of the confined region was defined as the midpoint between the carbonyl carbon atoms of TYR302 of cytochrome b and HIS135 of the ISP, and for model II, the midpoint between the Fe2 iron atom of Fe_2_S_2_ (see Figure 3) and the nearest of the two unprotonated oxygens of ${\text{Q}}^{•-}$ was used.

The ParseFEP plugin (Liu et al., 2012) of VMD was used to analyze the results of the FEP simulations and produce free energy curves using the Bennet acceptance ratio (BAR) estimator (Bennett, 1976) based on the combined results from forward and backward simulations.

## 3 Results and Discussion

The starting point of the present investigation was an earlier computational study (Husen and Solov’yov, 2016) of the dynamics and binding of molecular oxygen inside the $bc_{1}$ complex (PDB ID: 1ZRT (Berry et al., 2004)). From these earlier simulations, parts of simulated trajectories with O_2_ bound in a pocket near the Q_*o*_-site were extracted and used for the simulations and analysis presented here. First, we discuss the results from quantum chemical modeling of a region around the Q_*o*_-site with O_2_ bound, where we generate statistics of local spin densities and identify possible chemical reactions leading to superoxide production. Next, we utilize MD-based approaches to estimate the free energy due to such electron transfer processes. Finally, we estimate the possible superoxide production rate based on the free energy results.

### 3.1 Quantum Chemical Modeling of the Q_*o*_-Site

A total of 200 snapshots from MD simulations with O_2_ trapped close to the Q_*o*_-site of the $bc_{1}$ complex were randomly selected, and a 263 atom “quantum region” consisting of the local environment at the Q_*o*_-site (see Figure 3) was extracted and analyzed in QC calculations using Gaussian 09 (Frisch et al., 2013; Yanai et al., 2004; Rassolov et al., 2001) following a protocol described previously (Salo et al., 2017). The large number of snapshots was employed to extract ensemble statistics from the earlier MD simulations Husen and Solov’yov (2016) in a local minimum characterized by having O_2_ bound in the pocket near Fe_2_S_2_ depicted in Supplementary Figure S5 in the SM, while ${\text{Q}}^{•-}$ is bound at the Q_*o*_-site. The need for increased sampling is emphasized by the large variability in outcome from quantum chemical modeling observed earlier (Salo et al., 2017). The QC calculations presented here essentially consist of a geometry optimization using the CAM-B3LYP DFT method using the 6-31G basis set (Rassolov et al., 2001; Yanai et al., 2004) carried out for each extracted snapshot with backbone carbon atoms kept at fixed positions and thereby carrying the conformational variation sampled from MD simulations. The relatively limited 6-31G basis set has earlier been shown to model important properties of iron-sulfur clusters correctly (Szilagyi and Winslow, 2006; Barragan et al., 2015) and was chosen to support the inclusion of a rather large region at the Q_*o*_-site in the quantum model. The limitations of the basis set, lacking diffuse and polarization functions, should however be considered as a source of uncertainty in the results as also discussed below.

**FIGURE 4:**
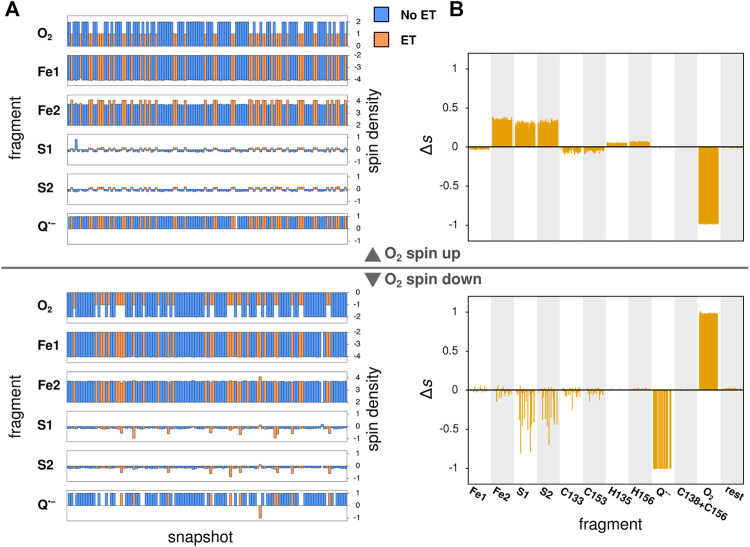
Local spin densities resulting from QC calculations on MD snapshots. **(A)** The local spin densities obtained from QC calculations on MD snapshots with O_2_ bound near the Q_*o*_-site. The snapshots where electron transfer to O_2_ has occurred, as determined by a change in O_2_ spin density, are indicated with orange color, while snapshots with no electron transfer are shown in blue. **(B)** The difference Δs between the local spin density in each snapshot where electron transfer is observed and the average of all snapshots with no electron transfer. The top half of the figure shows the results of QC calculations with O_2_ in the spin up state, while the botton shows results for the spin down state.

In its ground state, O_2_ is a triplet diradical, and the two unpaired electrons were modeled in both spin up and spin down configurations, where the unpaired electrons of the iron atom Fe2 of the Fe_2_S_2_-cluster (see Figure 3) are considered as spin up. The calculations successfully converged for 127 and 114 snapshots, when modeled with O_2_ spin up and O_2_ spin down, respectively. After geometry optimization, a spontaneous electron transfer to the bound O_2_ molecule was observed in some, but not all cases: as can be seen in Figure 4A, the spin density of O_2_ is either 2 (no charge transfer) or 1 (charge transfer occurred) for the spin up case and −2 and −1, respectively, for the spin down case. The sign of the spin densities in Figure 4A indicate the spin orientation of the fragment, so it can e.g. be observed that the anti-parallel spin between the iron atoms of Fe_2_S_2_ is maintained after the QC geometry optimization.

To identify the source of the electron transferred to the O_2_ molecule, Figure 4B shows the difference in local spin density between each of the studied snapshots, where electron transfer to O_2_ was observed, and the average of all snapshots with no electron transfer, i.e. the quantity:

$$\text{Δ}s_{i}=s_{i}-{\langle s\rangle }_{\text{no ET}},\;i\in \left\{\text{snapshots with ET}\right\}.$$ (5)

Here, $s_{i}$ is the local spin density calculated for a snapshot *i*, where electron transfer was observed, and ${\langle s\rangle }_{\text{no ET}}$ is the average of the local spin density among all snapshots, where no electron transfer was observed. In the case of O_2_ spin up, the electron is consistently transferred from the Fe_2_S_2_-cluster (primarily atoms Fe2, S1 and S2, see Figure 3), while in the spin down case, the electron comes either fully from the semiquinone anion or again from the Fe_2_S_2_-cluster, but this time primarily from the two sulfur atoms. These results confirm earlier findings that an electron can be transferred to O_2_ either from ${\text{Q}}^{•-}$ (model I) or from Fe_2_S_2_ (model II) and its coordinating amino acids (Salo et al., 2017). The extended statistics provided here clearly separates the possible reactions into two principal scenarios, corresponding to the two models studied through MD modeling in the following. A comparison of the initial geometries leading to different electronic configurations after QC calculations, shown in Supplementary Figure S2 in the SM, revealed that the position of the bound O_2_ molecule is important for electron transfer to happen, but no clear deciding geometric features of ${\text{Q}}^{•-}$ or the protein are immediately apparent.

**FIGURE 5:**
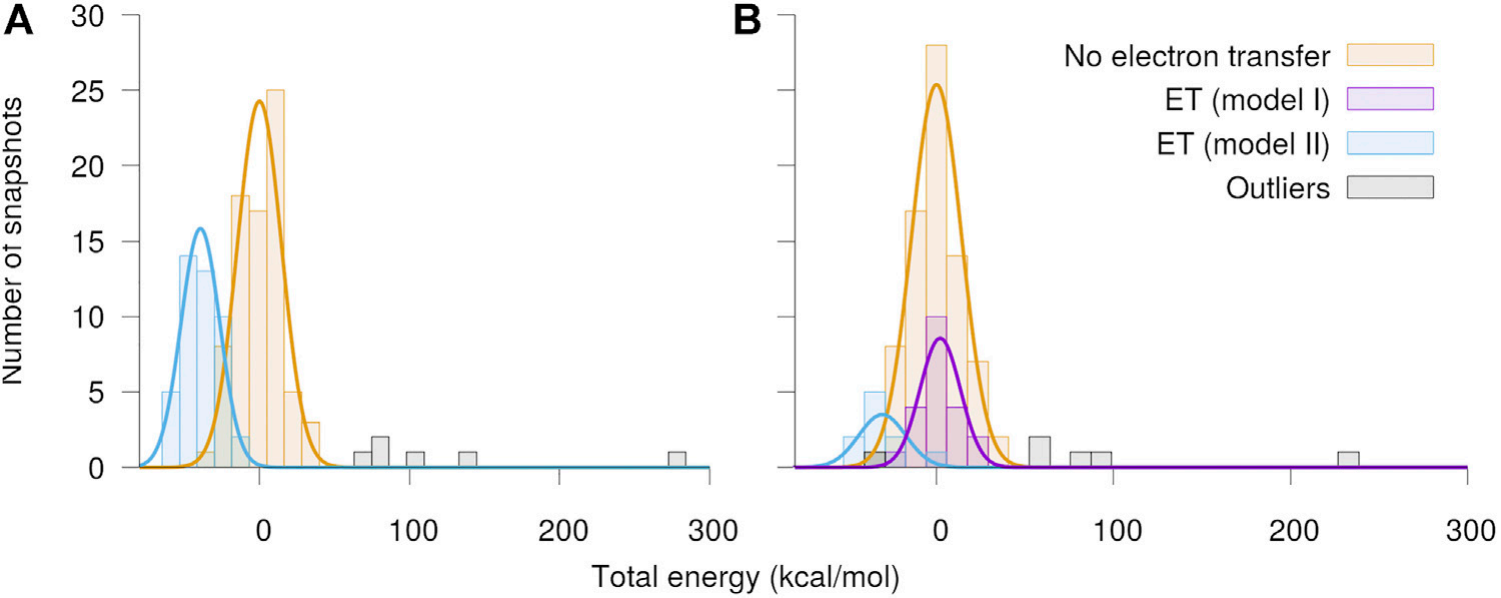
Distribution of total energy of the quantum region after QC geometry optimization. The calculations were performed separately with O_2_ assumed to be in the **(A)** spin up and **(B)** spin down state, respectively. The snapshots have been classified into cases with no electron transfer and electron transfer from either the semiquinone (model I) or the Fe_2_S_2_-cluster (model II) to O_2_. The energies are shown relative to the mean energy of the snapshots with no charge transfer.

The distributions of total energies of the modeled Q_*o*_-site of the $bc_{1}$ complex separated into cases with and without observed charge transfer events are shown in Figure 5. The mean energies and standard deviations of the separate distributions are listed in Table 1 and were used to draw the Gaussian fit functions presented in Figure 5. A small number of outlier cases, determined based on an energy threshold, fall well outside the main identified distributions. Various alternative electron transfers, mostly not involving the O_2_ molecule, were observed in the outlier cases. In the snapshots leading to electron transfer from Fe_2_S_2_^−^ to O_2_ (model II), the energy is noticeably lower than for snapshots with no electron transfer, while there is virtually no energy difference in case of electron transfer from ${\text{Q}}^{•-}$ (model I). The calculations indicated that both reactions are clearly allowed, when considering the quantum energetics of the Q_*o*_-site alone. In the following, classical MD methods are used to model effects of the local environment in the two model reactions, including binding of $O_{2}^{•-}$, $bc_{1}$ complex reorganization and the free energy of the combined reaction complex. This is in poor agreement with the experimental evidence, which essentially rules out Fe_2_S_2_ as an electron donor (Sun and Trumpower, 2003; Bleier and Dröse, 2013). The relatively small quantum region has, however, been isolated in our model, while the effects of the surroundings, i.e. the solvent or, in this case, the protein environment, is likely to play a significant role in electron transfer processes (Moser and Dutton, 1992; Moser et al., 1992; Miyashita et al., 2003).

**TABLE 1:** Energy statistics from the QC geometry optimizations. The table shows the numbers *N* of snapshots along with mean energies 〈E〉 and standard deviations σ, where no electron transfer or electron transfer according to model I, Eq. 1, or model II, Eq. 2, was observed following QC geometry optimization of randomly selected MD simulation snapshots. The energies are computed relative to the mean energy of snapshots with no electron transfer observed.

	O_2_ spin up			O_2_ spin down		
	N	〈E〉 (kcal/mol)	σ (kcal/mol)	N	〈E〉 (kcal/mol)	σ (kcal/mol)
No ET	77	0	14.730	77	0	14.088
ET (model I)	0			21	2.0507	11.399
ET (model II)	44	−39.333	12.906	10	−30.672	13.267
Outliers	6			6		

The size of the studied quantum region and the choice of basis set needed to be balanced due the high computational complexity of QC modeling methods. The modeled region was chosen with proximity to the O_2_ molecule bound near the Q_*o*_-site as the main criterion and matches the model studied in an earlier investigation (Salo et al., 2017). To assess the significance of the choice of cluster model on the results from QC modeling, all calculations were repeated using the same MD snapshots, but with the cluster model extended to include E295 from cytochrome b, which is likely to be an important interaction partner with QH_2_/ ${\text{Q}}^{•-}$, along with its immediate neighbors, V293 and P294 (Lee et al., 2011; Crofts et al., 2017). The results are shown in Supplementary Figures S3, S4 as well as Supplementary Table S2 of the SM. In this case, only three snapshots out of 109 led to the charge transfer described by model I, while the model II reaction remains prevalent in the extended model. The high sensitivity to the choice of included amino acid residues indicates that a limited cluster model is insufficient to reliably and quantitatively model electron transfer processes in a highly complex protein environment. Furthermore, the use of the limited basis set, 6-31G, also introduce uncertainties, but these are likely overshadowed by the uncertainties due to the choice of quantum region. In the present investigation, the results from quantum chemical modeling are ultimately used in an exploratory fashion to identify possible reactions leading to superoxide production: while the initial motivation was the hypothesis that a highly reactive semiquinone anion could react with an oxygen molecule, the frequent observation of electron transfer from Fe_2_S_2_^−^ led us to include both models in the further free energy calculations.

Aside from the redox states of the potential reaction partners at the Q_*o*_-site, the protonation of the bound semiquinone was also tracked in the extended quantum calculations to test for possible proton transfer back to ${\text{Q}}^{•-}$. The second proton transfer after binding of QH_2_ at the Q_*o*_-site is generally believed to go to E295 (Crofts et al., 2017), either directly or through Y147 as an intermediate (Barragan et al., 2016), so in the extended model, which includes E295, it is conceivable that the proton would be transferred back, if the anionic semiquinone at the Q_*o*_-site is unstable. None of the studied snapshots led to proton transfer from E295 or Y147, but in around 4% of cases, proton transfer from Y302 to ${\text{Q}}^{•-}$ was observed. In the majority of cases, ${\text{Q}}^{•-}$ thus remained fully deprotonated, so the existence of a local minimum with anionic semiquinone bound at the Q_*o*_-site remains plausible.

### 3.2 Unbinding of Superoxide

**FIGURE 6:**
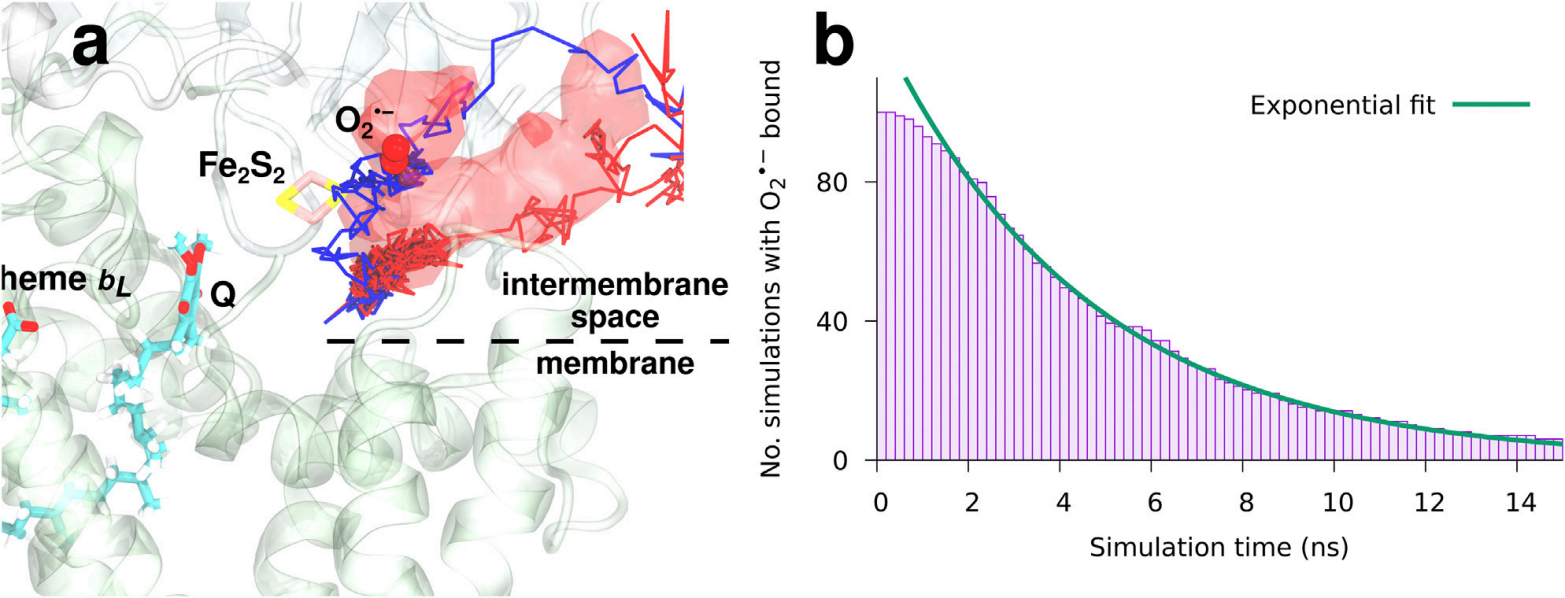
The dynamics and binding of a newly formed superoxide (model I). ${\text{O}}_{\text{2}}^{•-}$ stays bound for a while in the binding pocket near the Q_*o*_-site, but eventually escapes into the bulk water. **(A)** superoxide escapes into the intermembrane space. The red regions indicate the averaged localization of O_2_ during all 100 simulated unbinding events. Two example trajectories are shown with red and blue colors. **(B)** a histogram of the number $N_{b}\left(t\right)$ of simulations that still has ${\text{O}}_{\text{2}}^{•-}$ bound after a specified simulation time along with a fit of an exponential model, Eq. 6, with a characteristic binding time of τ=4.5 ns.

To study the binding and dynamics of superoxide after an electron transfer event at the Q_*o*_-site, a set of 100 MD simulations starting with $O_{2}^{•-}$ bound in place of O_2_ in the pocket depicted on Supplementary Figure S5 in the SM were carried out for each of the two models following an approach previously used to model O_2_ binding and unbinding in proteins (Husen et al., 2019). All simulations were continued until $O_{2}^{•-}$ unbinding was observed. $O_{2}^{•-}$ is indeed able to bind in the same pocket as O_2_ near the Q_*o*_-site, where it stays for a while before unbinding as illustrated in Figure 6 and Supplementary Figure S5C for model I. While the neutral O_2_ enters the binding pocket from the membrane (Husen and Solov’yov, 2016), $O_{2}^{•-}$ was found to consistently escape directly into the water phase on the positive side of the membrane, i.e. the intermembrane space in case of mitochondria, in all 100 simulations for each model. Two example trajectories of $O_{2}^{•-}$ unbinding are shown in Figure 6A with red and blue lines, and the red surface is an isosurface of the $O_{2}^{•-}$ density averaged over all 100 unbinding simulations, i.e. indicating the general localization of the anion during the simulations.

The observed occupancy of $O_{2}^{•-}$ in the binding position near the Q_*o*_-site of the $bc_{1}$ complex, defined as the fraction of simulations that still has $O_{2}^{•-}$ bound, is shown in Figure 6B as a function of simulation time for model I. A fit of an exponential model,

$$N_{b}\left(t\right)=N_{0}e^{-\frac{t}{\tau }},$$ (6)

reveals a characteristic binding time of τ=4.5  ns for model I and τ=1.6  ns for model II (see Supplementary Figure S6 in the SM). The fact that $O_{2}^{•-}$ remains briefly bound points to the existence of a local minimum in the product state of the electron transfer reaction, which puts the free energy analysis in the following on a stronger footing. For both models, the superoxide anion consistently leaves into the positive side of the membrane (the intermembrane space, in case of mitochondria), when it unbinds. This is unlike the initial binding of the neutral (and non-polar) O_2_ molecule, which arrives through the membrane (Husen and Solov’yov, 2016). The finding clearly indicates that mitochondrial superoxide production originating from the $bc_{1}$-complex will primarily emit superoxide into the intermembrane space.

### 3.3 Reorganization Energy From Classical MD Simulations

In order to construct free energy diagrams of possible electron transfer processes that could lead to superoxide production at the Q_*o*_-site of the $bc_{1}$ complex, a number of MD simulations with O_2_ bound in the binding pocket near Fe_2_S_2_ were carried out to sample the potential energy surfaces in the reactant and product states; in particular, the potential energy difference, ΔU , for transferring the system from the reactant to the product charge state for a given set of nuclear coordinates is sampled. The sampled energy histograms are then used to calculate the free energy curves with ΔU serving as the reaction coordinate. The simulations and analysis were carried out for both of the two models of superoxide formation, differing in the source of the electron transferred to O_2_. For both models, simulations from an earlier study (Husen and Solov’yov, 2016) were used in the analysis as the reactant state with O_2_ bound near the Q_*o*_-site in the presence of ${\text{Q}}^{•-}$, i.e. after the initial electron transfer from QH_2_ in the Q-cycle. Simulations of the product states were then set up with O_2_ converted to $O_{2}^{•-}$ and either ${\text{Q}}^{•-}$ oxidized to Q (model I) or Fe_2_S_2_^−^ oxidized to Fe_2_S_2_ (model II) at the Q_*o*_-site.

**FIGURE 7:**
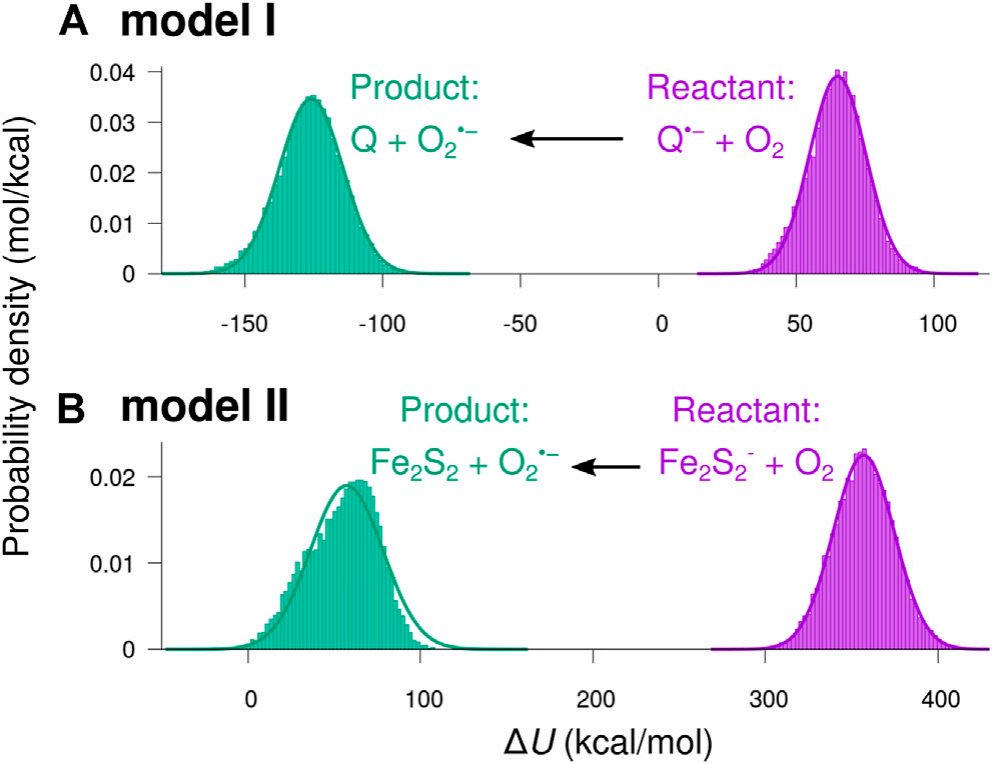
Distributions of the potential energy shift from reactant to product charge state. The potential energy difference ΔU for going from the reactant to product charge state for a given set of atomic coordinates was sampled through MD simulations separately in the product and reactant states for the two studied reactions, Eqs 1, 2, where the electron received by O_2_ originates from **(A)** semiquinone (model I) or from **(B)** the Fe_2_S_2_-cluster (model II), respectively.

Figure 7 shows histograms of the energy difference ΔU sampled from MD simulations. Specifically, ΔU is obtained by calculating the potential energy in a given simulation snapshot, which defines a set of atomic coordinates, ${\vec{x}}_{1},\ldots ,{\vec{x}}_{N}$ , by separately assigning atomic partial charges and model parameters describing the system in the reactant and product states:

$$\text{Δ}U\left({\vec{x}}_{1},\ldots {\vec{x}}_{N}\right)=U_{\text{product}}\left({\vec{x}}_{1},\ldots {\vec{x}}_{N}\right)-U_{\text{reactant}}\left({\vec{x}}_{1},\ldots {\vec{x}}_{N}\right).$$ (7)

The smooth curves in Figure 7 indicate normal distributions based on the statistical mean values, 〈ΔU〉, and standard deviations, σ, from the sampled ΔU distributions:

$$\rho \left(\text{Δ}U\right)=\frac{1}{\sqrt{2\pi \sigma^{2}}}e^{-\frac{{\left(\text{Δ}U-\langle \text{Δ}U\rangle \right)}^{2}}{2\sigma^{2}}}.$$ (8)

Except for a small skewness for the product state in model II, the sampled ΔU appears to be normal distributed, indicating that the sampling is carried out within a single local minimum of the free energy landscape.

The potential energy difference is a function of atomic coordinates alone and is therefore suitable as a reaction coordinate describing the mechanical reorganization accompanying the modeled electron transfer process (Miyashita and Go, 2000; Miyashita et al., 2003). By sampling ΔU fluctuations in local minima around both product and reactant states, a set of free energy curves can be constructed by taking the logarithm of the densities of states (Warshel, 1982; Miyashita et al., 2003) shown in Figure 7:

$$G=-k_{B}T\;\text{ln}\left[\rho \left(\text{Δ}U\right)\right]+\text{constant}.$$ (9)

Assuming a normal distribution of ΔU, Eq. 8, the free energy curve acquires a parabolic trend:

$$G=\frac{k_{B}T}{2\sigma^{2}}{\left(\text{Δ}U-\langle \text{Δ}U\rangle \right)}^{2}+G_{0}.$$ (10)

**FIGURE 8:**
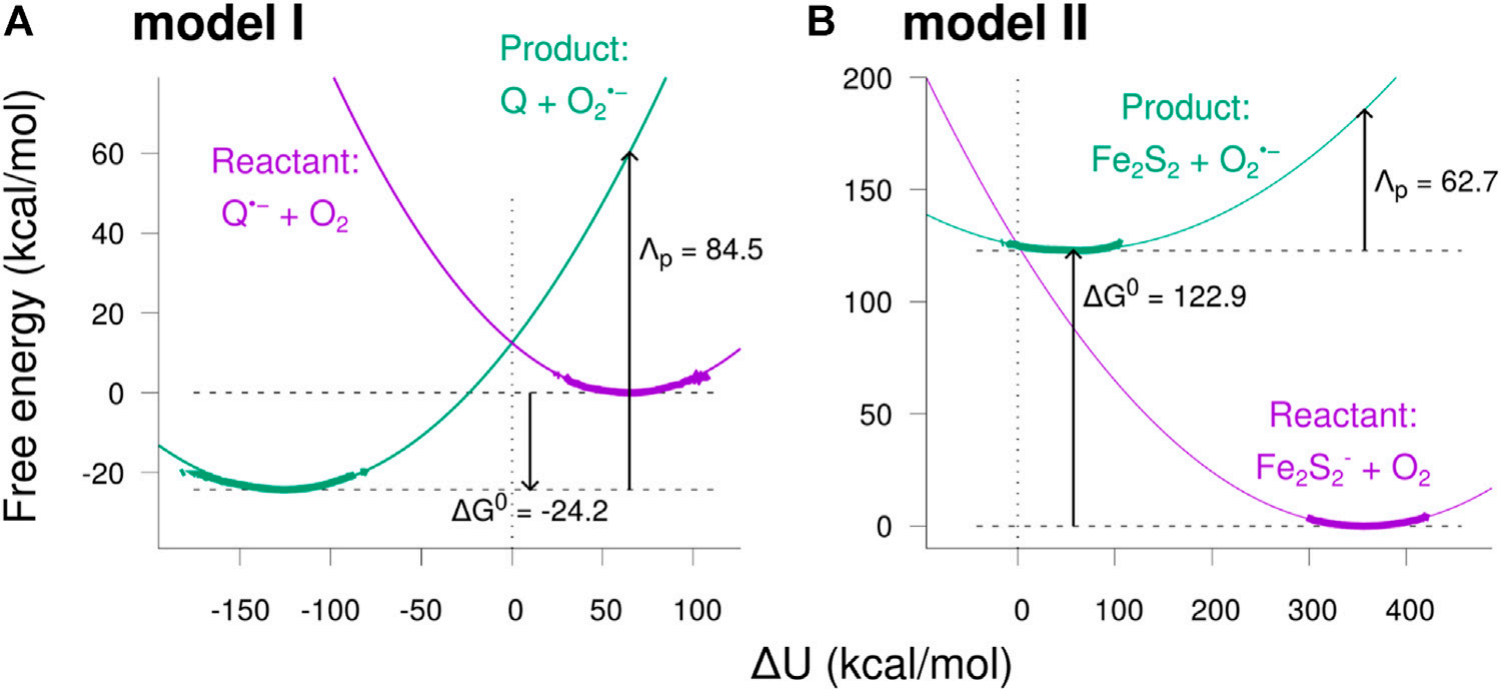
Free energy diagrams for the two model reactions. The free energy was calculated as a function of the potential energy shift ΔU sampled in the MD simulations (see Figure 7) in the reactant and product states. **(A)** results for model I, **(B)** results for model II. The thick curves indicate the free energies sampled directly from the simulations, while the thinner curves are the extrapolated parabolas, obtained using Eq. 10. The free energy difference $\text{Δ}G^{0}$ between the reactant and product state minima and the reorganization energy ${\text{Λ}}_{p}$ calculated in the product state are indicated in the figure, both values in kcal/mol.

The resulting free energy curves for the studied electron transfer processes are depicted in Figure 8. The figure shows the free energy calculated from the sampled histograms in Figure 7 by using Eq. 9 with thick lines, while extrapolated parabolas obtained through Eq. 10 are shown with thin lines. The free energy of the product state was shifted to make the two curves intersect at ΔU=0 (Miyashita et al., 2003). $\text{Δ}G^{0}$ shown in the figure is the difference between the minima of the two free energy curves, and the reorganization energy, ${\text{Λ}}_{p}$ , is the energy required to reorganize the atoms of the local environment from their equilibrium position in the product state, i.e. after electron transfer, to correspond to the equilibrium positions in the reactant state. The reorganization energy ${\text{Λ}}_{p}$ and the similarly defined ${\text{Λ}}_{r}$ for moving atoms in the reactant state to the equilibrium positions in the product state were observed to differ (see Table 2), indicating that the medium fluctuations do not globally follow Gaussian statistics (LeBard and Matyushov, 2010; Martin and Matyushov, 2012; Martin et al., 2013; Matyushov, 2013). The product state value, ${\text{Λ}}_{p}$ , was used, as the two parabolas intersect at the product state equilibrium when ${\text{Λ}}_{p}=-\text{Δ}G^{0}$ , leading to maximal overlap integral between the reactant and the product harmonic oscillator states (Moser et al., 1992; Moser and Dutton, 1992).

**TABLE 2:** Free energy parameters and estimated rates obtained from simulations. $\text{Δ}G_{\text{reorg}}^{0}$ is the free energy difference between the reactant and product state equilibria estimated from the two-state reorganization energy calculations, and ${\text{Λ}}_{p}$ and ${\text{Λ}}_{r}$ are the corresponding reorganization energies estimated in the product and reactant state, respectively (see Figure 8). $\text{Δ}G_{\text{FEP}}^{0}$ is the free energy change estimated through FEP simulations. $k_{\text{et}}$ is the electron transfer rate estimate obtained using $\text{Δ}G_{\text{FEP}}^{0}$, ${\text{Λ}}_{p}$ and Eq. 13.

	$\text{Δ}G_{\text{reorg}}^{0}$	${\text{Λ}}_{p}$	${\text{Λ}}_{r}$	$\text{Δ}G_{\text{FEP}}^{0}$	$k_{\text{et}}$
	(kcal/mol)	(kcal/mol)	(kcal/mol)	(kcal/mol)	S^−1^
Model I	−24.5	84.5	107.9	−21.6	$6.1\times 10^{5}$
Model II	122.9	62.7	88.3	162.7	$1.0\times 10^{-98}$

The free energy diagrams in Figure 8 and the parameters Λ and $\text{Δ}G^{0}$ could be used to estimate the rate of electron transfers leading to superoxide production using the Marcus theory (Marcus and Sutin, 1985; Page et al., 1999). However, as the sampled ΔU distributions are rather far from directly overlapping, quite extensive extrapolation is involved, which leads to a high degree of uncertainty in the estimated values of $\text{Δ}G^{0}$ and ${\text{Λ}}_{p}$. To alleviate this uncertainty, a different approach of free energy estimation was employed.

### 3.4 Free Energy Perturbation Calculations

Instead of connecting the product and reactant states in a single step, an alchemical free energy perturbation (Dixit and Chipot, 2001; Miyashita et al., 2003) (FEP) approach was employed as a second attempt of identifying the free energy change due to an electron transfer to O_2_. In this approach, the electrostatic interactions involving O_2_/$O_{2}^{•-}$ and ${\text{Q}}^{•-}$ /Q for model I and Fe_2_S_2_^−^/Fe_2_S_2_ (including the coordinating amino acids and SER158 of the ISP) for model II were gradually modulated by a parameter λ during MD simulations, where $\lambda =\lambda_{1},\lambda_{2},\ldots \lambda_{N}$ steps between 0 and 1 in a series of simulation windows. First, the electrostatic interactions were turned off in the reactant state. Then, they were turned back on in the product state charge configuration. Only electrostatic interactions were modulated, as no atoms or bonds are introduced or removed in either of the studied reactions, and the van der Waals interactions were assumed to be the same for the two redox states. The total free energy difference between reactant and product state was then calculated by adding up the contributions from each λ-step:

$$\text{ΔΔ}G_{i}=-k_{B}T\text{ln}\langle e^{-\frac{\text{Δ}U_{i}}{k_{B}T}}\rangle ,\;\;\text{Δ}G^{0}=\underset{i}{\sum }\text{ΔΔ}G_{i}.$$ (11)

Here, $\text{ΔΔ}G_{i}$ is the change in free energy when stepping the λ parameter from $\lambda_{i}$ to $\lambda_{i+1}$ , and $\text{Δ}G^{0}$ is the total change in free energy between the reactant and the product states. The sampled variable, $\text{Δ}U_{i}$ , is the difference in potential energy due to changing λ from $\lambda_{i}$ to $\lambda_{i+1}$ for a given set of atomic coordinates, sampled in a simulation with a force field corresponding to $\lambda =\lambda_{i}$. The FEP transformation is carried out both in forward and backward mode, and the results are combined to produce a single estimate of the free energy change as a function of λ by using the BAR estimator (Bennett, 1976) in the ParseFEP plugin (Liu et al., 2012) of VMD (Humphrey et al., 1996). The results of the individual forward and backward transformations and the BAR estimates are summarized in Supplementary Tables S3, S4 in the SM. The combined results of the free energy calculations are shown in Supplementary Figure S7. The free energy due to the discharging (FEP1) and charging (FEP2) transitions have been connected in the figure, such that the total end-to-end free energy difference in the diagram reflects the total free energy change, $\text{Δ}G^{0}$ , of the electron transfer reaction. The results are shown for three settings of the simulation length per λ-window. The results are further broken down in Supplementary Figures S10–S21 of the SM, where distributions of the sampled $\text{Δ}U_{i}$ for each λ step are shown along with the separate free energy curves for the forward and backward simulations. When 1 ns or 2 ns simulation time per λ step is used, the sampled $\text{Δ}U_{i}$ appear as normal distributed, and the forward and backward results are generally in good correspondence with discrepancies up to 5 kcal/mol (see Supplementary Tables S3, S4). The effects of the artificial restraints keeping the O_2_ molecule from leaving its binding pocket are assumed to be minor, as the O_2_ remains naturally in the bound position during most of the simulations as further discussed in the SM.

The electron transfer from ${\text{Q}}^{•-}$ (model I) leads to a significant decrease in free energy of around 20 kcal/mol, indicating a favorable reaction, while the reaction in model II has a quite high free energy cost of 160 kcal/mol. The reaction free energies are summarized in Table 2. The free energy change for the reaction in model I is roughly similar to the result from the reorganization energy sampling (Figure 8), while the discrepancy is somewhat bigger for model II. In the latter case, however, both models agree that the free energy cost is so large that the reaction is virtually impossible. The gradual change of potential in the free energy perturbation approach makes sure that the ΔU histograms for a forward and backward step are always overlapping, leading to a much higher confidence in the resulting free energy difference. However, barriers in the free energy landscape and the reorganization energy cannot be obtained using the free energy perturbation method, since an alchemical and unphysical route is used to sample the energy profile.

### 3.5 Rate of Superoxide Production

The obtained free energy shifts and reorganization energies can be used to estimate the possible rates of the modeled electron transfer processes. Marcus theory (Marcus and Sutin, 1985; Miyashita et al., 2003) predicts the following form of the electron transfer rate constant:

$$k_{\text{et}}\;=\;\frac{2\pi }{\hbar }|T_{AB}{|}^{2}\frac{1}{\sqrt{4\pi \text{Λ}k_{B}T}}\text{exp}\left(-\frac{{\left(\text{Λ}+\text{Δ}G^{0}\right)}^{2}}{4\lambda k_{B}T}\right),$$ (12)

where $\left|T_{AB}\right|$ is the electronic coupling constant between the reactant and product states, Λ is the reorganization energy, and $\text{Δ}G^{0}$ is the total change in Gibbs free energy between the minima of the two states. $\text{Λ}={\text{Λ}}_{p}$ and $\text{Δ}G^{0}$ can be taken from the reorganization and FEP calculations, respectively. Meanwhile, Dutton and Moser (Moser and Dutton, 1992; Moser et al., 1992) found the following empirical relationship for electron transfers in biomolecules:

$${\text{log}}_{10}k_{\text{et}}\;=\;15-0.6R-3.1\frac{{\left(\text{Δ}G+\text{Λ}\right)}^{2}}{\text{Λ}},$$ (13)

where *R* is the edge-to-edge distance between donor and acceptor in Å, energies are assumed in eV, and the electron transfer rate is in inverse seconds. In the reactant state simulations, the distance between donor and acceptor in model I (the head group of ${\text{Q}}^{•-}$ and the O_2_ molecule) fluctuates between 2 and 10 Å. Taking $\text{Λ}={\text{Λ}}_{p}$ and $\text{Δ}G^{0}=\text{Δ}G_{\text{FEP}}^{0}$ and applying Eq. 13 individually to 892 snapshots at 10 ps intervals from an O_2_ binding event in a simulated trajectory in the reactant state leads to average electron transfer rates of $6.1\times 10^{5}\;s^{-1}$ for model I as listed in Table 2. The results for model II indicates that the electron transfer from Fe_2_S_2_^−^ to O_2_ is virtually impossible.

The estimated electron transfer rates correspond to the state, where semiquinone and O_2_ are simultaneously present at the Q_*o*_-site of the $bc_{1}$ complex. The total superoxide production rate, while the $bc_{1}$ complex is in the semiquinone state, taking into account binding and unbinding of oxygen molecules can be estimated as:

$$k_{{\text{O}}_{2}^{•-}|\text{SQ}}\;=\;k_{\text{bind}}\frac{k_{\text{et}}}{k_{\text{et}}+k_{\text{unbind}}},$$ (14)

where $k_{\text{bind}}$ and $k_{\text{unbind}}$ are the rates of binding and unbinding of O_2_ at the binding pocket near the Q_*o*_-site. A previous investigation (Husen and Solov’yov, 2016) estimated the binding and unbinding rates under physiological oxygen concentrations as $k_{\text{bind}}=2\times 10^{5}\;{\text{s}}^{-1}$ and $k_{\text{unbind}}=10^{8}\;{\text{s}}^{-1}$ , which leads to a superoxide production rate in the semiquinone state for model I of:

$$k_{{\text{O}}_{2}^{•-}|\text{SQ}}\;≃\;1.2\times 10^{3}\;{\text{s}}^{-1}.$$ (15)

This is a very rough order of magnitude estimate, as e.g. using instead the reorganization energy estimate from the reactant state, $\text{Λ}={\text{Λ}}_{r}$ , yields a two orders of magnitude lower electron transfer rate, but the results clearly indicate that the reaction described in model I is plausible, while the electron transfer from Fe_2_S_2_ in model II is prohibited due the local protein environment. An experimental study on rat mitochondria (Quinlan et al., 2011) estimated values of $k_{{\text{O}}_{2}^{•-}|\text{SQ}}$ ($k_{9}$ in the referenced study (Quinlan et al., 2011)) ranging from 1 to 40 ${\text{s}}^{-1}$ by fits of different kinetic models to experimental superoxide production data, which taking into account the uncertainties, is qualitatively consistent with our computational findings. It should be noted that the rate estimations shown here assume that the $bc_{1}$ complex is in a state of the Q-cycle that has semiquinone bound at the Q_*o*_-site. This will most likely only be the case a small fraction of the time (one estimate (Crofts et al., 2006) puts this fraction at $x_{\text{SQ}}≃4\times 10^{-8}$), so in reality the superoxide production rate per $bc_{1}$ complex will be much lower. This is not so surprising, as under normal conditions the mitochondrial ROS production should be low.

## 4 Conclusion

Quantum chemical modeling confirmed earlier findings (Salo et al., 2017) of two main electron transfer processes leading to superoxide formation at the Q_*o*_-site of the $bc_{1}$ complex, which were studied as models I and II, where an electron is donated by either the semiquinone anion, ${\text{Q}}^{•-}$, at the Q_*o*_-site or the iron-sulfur cluster, respectively. The more extended statistics in the present study clearly separates the studied snapshots into cases corresponding to the two electron transfer models as well as cases, where no electron transfer takes place. The shift in energy between the cases with the electron at the donor and acceptor, respectively, indicates a notable decrease in energy for model II, while the electron transfer from ${\text{Q}}^{•-}$ in model I leads to a slight increase. The data from QC calculations alone thus suggests that both reactions are possible, and the model II reaction is rather favorable, which is in contrast to the expected high redox potential of the Fe_2_S_2_-cluster. However, including the effects of reorganization of the local protein environment changes this picture dramatically: the free energy contributions due to the local environment were estimated by two separate methods based on classical all-atom MD simulations, leading to values of the free energy difference between reactant and product states of the studied reactions as well as the corresponding reorganization energies. The free energy contribution obtained through modeling of the entire complex showed that the effects of the local environment is critical. Essentially, the electron transfer from Fe_2_S_2_ (model II) is virtually impossible, as expected, while the electron transfer from ${\text{Q}}^{•-}$ could happen at low rates. The calculated electron transfer rates are at best rough order of magnitude estimates, but are in reasonable correspondance with available experimental values (Quinlan et al., 2011) and provide evidence pointing to semiquinone at the Q_*o*_-site of the $bc_{1}$ complex as a source of mitochondrial ROS production. The computational model could further be used to model other potential superoxide production mechanisms and to study the effects of e.g. mutations on ROS production rates to further connect the computational results with experimental evidence. Additionally, it is found that superoxide produced at the Qo-site will exclusively escape to the intermembrane space of mitochondria.

## Data Availability

The original contributions presented in the study are included in the article/Supplementary Material, further inquiries can be directed to the corresponding author.
