# Supplementary material for: Modeling the Energy Landscape of Side Reactions in the Cytochrome bc1 Complex
Source: Front Chem. 2021 May 19;9:643796. doi: 10.3389/fchem.2021.643796 (PMC8170094; doi:10.3389/fchem.2021.643796)
Supplement: Supplementary file 1 [file DataSheet1.pdf]

frontiers

Supplementary Material

SUPPLEMENTARY METHOD FIGURES

Table S1 summarizes the molecular dynamics simulations used to analyze the reorganization energy related to electron transfer and superoxide unbinding. Fig. S1 summarizes the set of simulation carried out in the free energy perturbation calculations.

	Type	Number of $O_2/O_2^{\bullet-}$	Q_o-site	Ensemble	Simulation time (ns)	Repetitions
**a**	Equilibration	None	QH_2, Fe_2S_2	NPT	400	1
	Simulation	None	QH_2, Fe_2S_2	NVT	360	1
**b**	Equilibration	165	$Q^{\bullet-}$, $Fe_2S_2^{-1}$	NPT	3	1
	Simulation	165	$Q^{\bullet-}$, $Fe_2S_2^{-1}$	NVT	370	2
**c**	Model I	1	Q, $Fe_2S_2^{-1}$, $O_2^{\bullet-}$	NVT	1 – 31	100
	Model II	1	$Q^{\bullet-}$, Fe_2S_2, $O_2^{\bullet-}$	NVT	1 – 15	100

Table S1. The MD simulations used for the analysis in the present investigation. A combination of earlier and new MD simulations of various states of the bc_1 complex is used. **a**) The model of the membrane embedded bc_1 complex was initially constructed and equilibrated in an earlier study Barragan et al. (2015) focusing on the binding of QH_2 in the Q_o-site. **b**) Building upon the equilibrated structure form **a**, the dynamics and binding of O_2 in the bc_1 complex in a state with $Q^{\bullet-}$ bound in the Q_o-site was modeled in another study Husen and Solov'yov (2016), **c**) In the present investigation, segments of the simulated trajectories from **b** with O_2 bound in a pocket near the Q_o-site were extracted and used for analysis and for setting up a number of new simulations studying the unbinding of $O_2^{\bullet-}$ after a putative electron transfer according to model I and II, respectively.

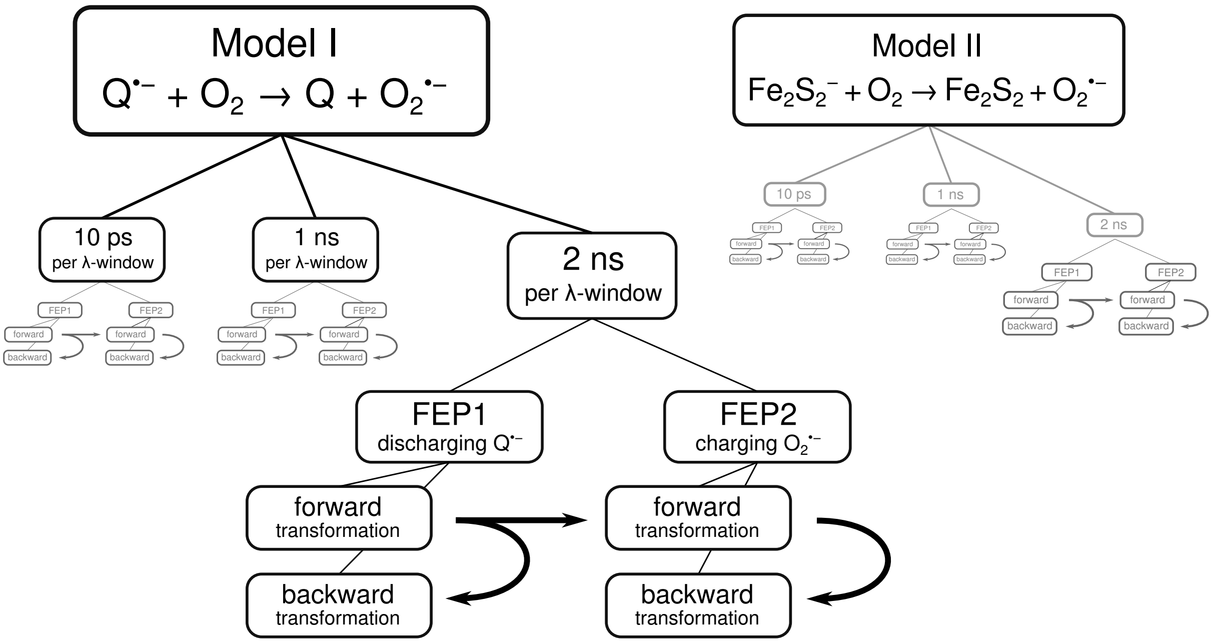

Figure S1. Diagram of the 24 performed FEP simulations: Two models for the electron transfer process, see Fig. 2, were modeled by dividing each of them into a discharging (FEP1) and a charging (FEP2) process. Each subprocess was studied through both a forward and a backward simulation. All of this was repeated for three settings of the simulation time per coupling parameter window.

SUPPLEMENTARY RESULTS

Geometric comparison of QC snapshots

In order to elucidate the effect of the geometry of the Q_o-site and its ligands on possible electron to O_2, a comparison was made between initial geometries of quantum chemically modeled region in snapshots that were observed to lead to different outcomes in terms of electron transfer (see Fig. S2). No obvious effect of geometric features of the protein or the semiquinone anion can be seen, but electron transfer to O_2 appear to require the O_2 molecule to be confined to a small region near Fe_2S_2 and the head group of of $Q^{\bullet-}$.

Extended quantum region

The QC calculations presented in Figs. 4 and 5 and Table 1 were repeated with the quantum cluster model (Fig. 3) extended to include the residues V293, P294 and E295 of cytochrome b. The results of the extended QC model are shown in Figs S3 and S4 and Table S2. With the extended model, the electron transfer

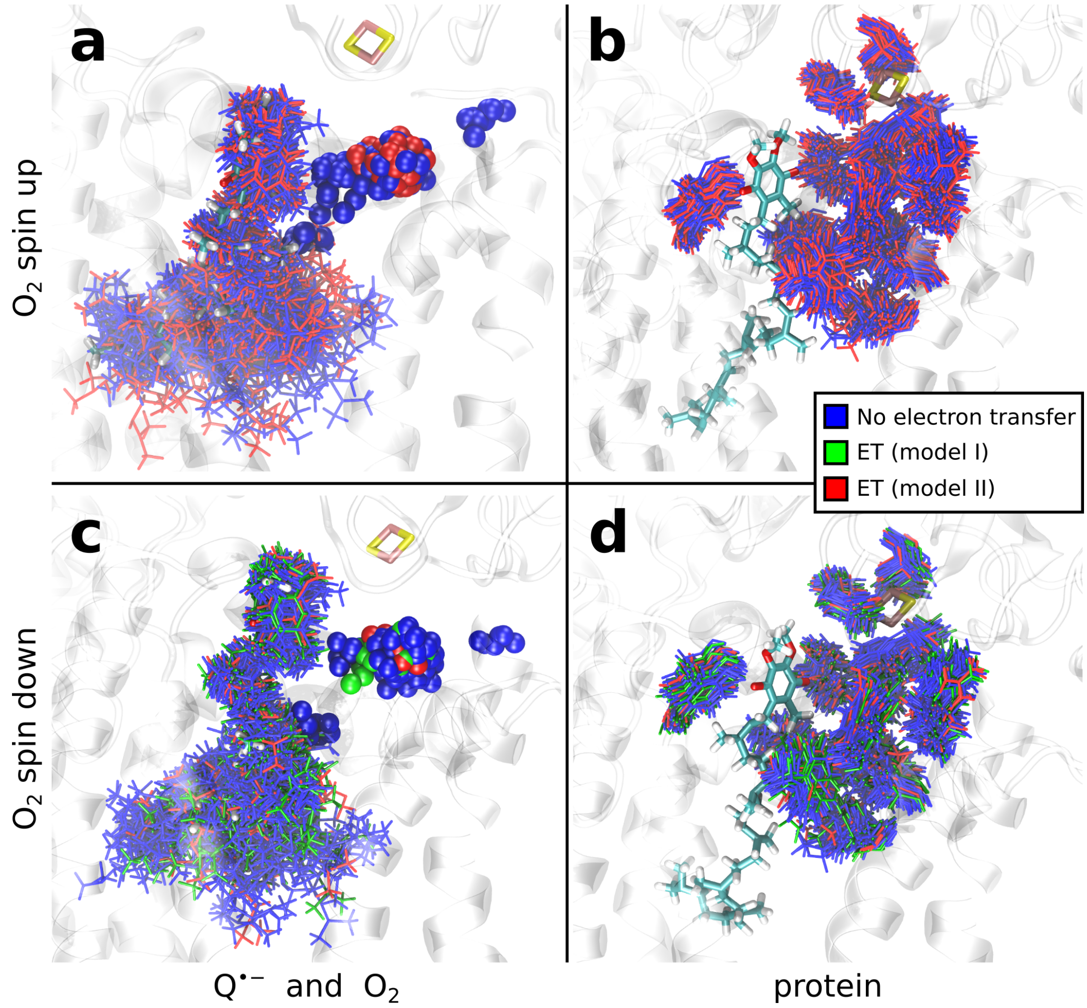

Figure S2. Geometric comparison of snapshots depending on outcome in QC calculations. The figure shows overlaid initial geometries of different parts of the cluster model used in the primary quantum calculations colored by the outcome in terms of observed electron transfer to O_2. **a** and **c** show the variation in the semiquinone ($Q^{\bullet-}$) and O_2 geometries in caclulations with O_2 modeled as spin up or spin down, respectively, while **b** and **d** show the same for the amino acid residues included in the quantum model.

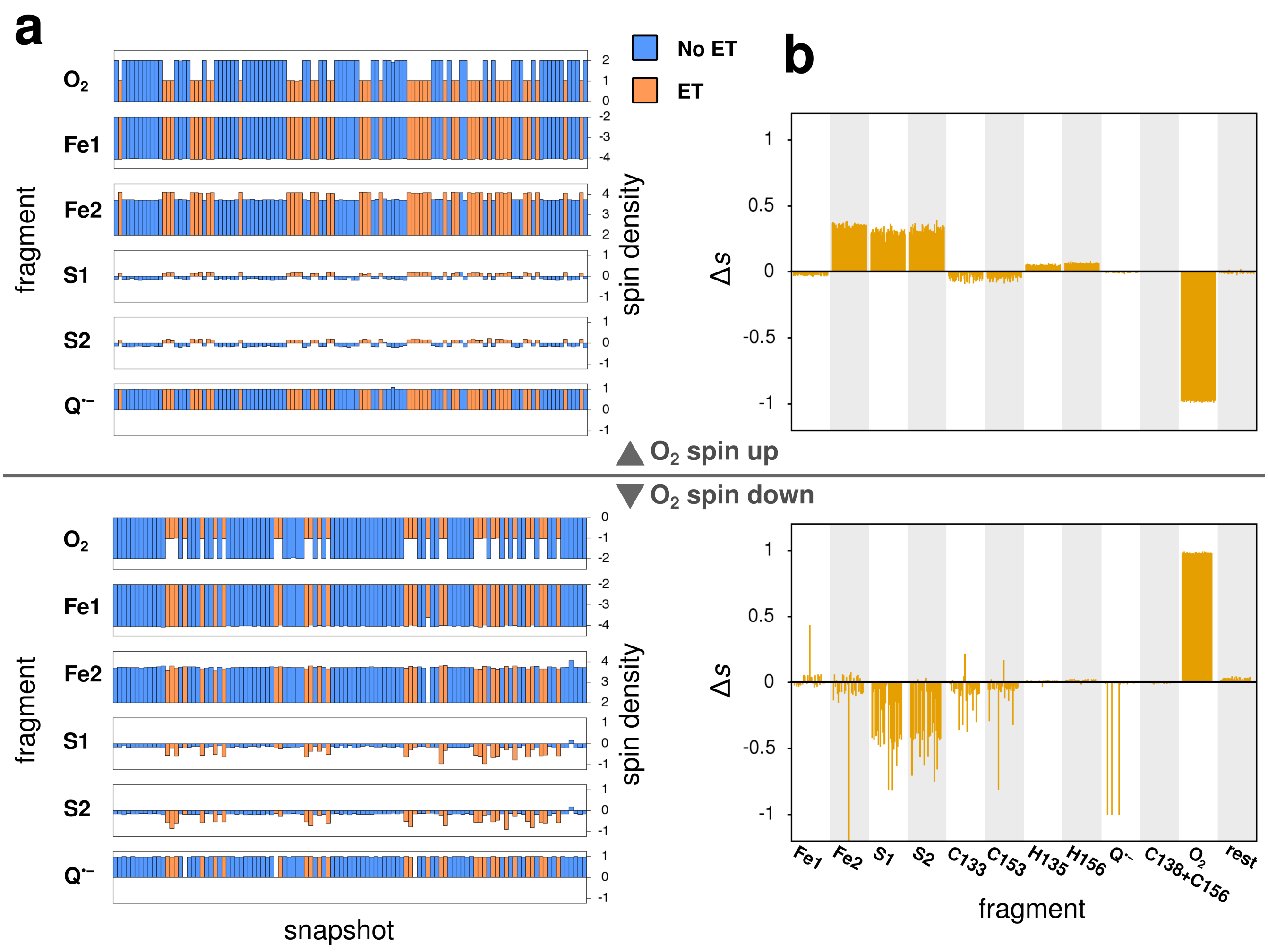

Figure S3. Local spin densities resulting from QC calculations on MD snapshots using the extended cluster model. a: The local spin densities obtained from QC calculations on MD snapshots with O_2 bound near the Q_o-site. The snapshots where electron transfer to O_2 has occurred, as determined by a change in O_2 spin density, are indicated with orange color, while snapshots with no electron transfer are shown in blue. **b:** The difference Δs between the local spin density in each snapshot where electron transfer is observed and the average of all snapshots with no electron transfer. The top half of the figure shows the results of QC calculations with O_2 in the spin up state, while the botton shows results for the spin down state.

from semiquinone to O_2 (model I) was only observed in three snapshots. This shows that the method is highly sensitive to the choice of model, although the inclusion of more residues does not change the overall conclusion that pure QC calculations indicate two possibilities for electron transfer. In the main paper, we include the results of the smaller quantum region, as this model has better statistics of the electron transfer events.

As E295 is believed to receive the second proton from QH_2, either directly or indirectly through Y147 (Crofts et al., 1999; Postila et al., 2013; Barragan et al., 2016), including the residue in quantum chemical modeling can also serve as a test of the stability of the anionic semiquinone in the Q_o-site. Out of 118 (109) completed calculations, $Q^{\bullet -}$was observed to receive a proton in 4 (6) cases, when O_2 was modeled in the spin-up (spin-down) state, i.e. it remained in anionic form in at least 95% of cases. This result indicates that the semiquinone anion exists at least as a metastable state at the Q_o-site.

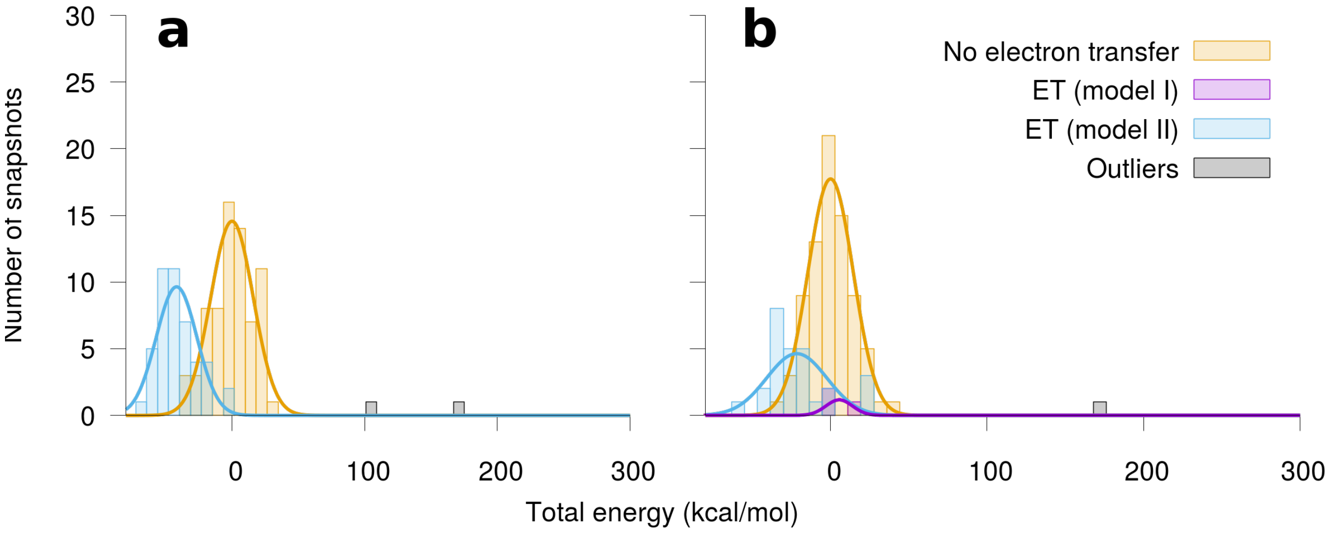

Figure S4. Distribution of total energy of the quantum region after QC geometry optimization in extended model. The calculations were performed separately with O_2 assumed to be in the (**a**) spin up and (**b**) spin down state, respectively. The snapshots have been classified into cases with no electron transfer and electron transfer from either the semiquinone (model I) or the Fe_2S_2-cluster (model II) to O_2. The energies are shown relative to the mean energy of the snapshots with no charge transfer.

	O_2 spin up			O_2 spin down		
	N	$\langle E \rangle$ (kcal/mol)	σ (kcal/mol)	N	$\langle E \rangle$ (kcal/mol)	σ (kcal/mol)
No ET	71	0	16.060	78	0	14.487
ET (model I)	0			3	5.4851	8.512
ET (model II)	45	-41.771	15.371	27	-21.615	19.219
outliers	2			1		

Table S2. Energy statistics from the QC geometry optimizations in extended model. The table shows the numbers N of snapshots along with mean energies $\langle E \rangle$ and standard deviations σ, where no electron transfer or electron transfer according to model I, Eq. (1), or model II, Eq. (2), was observed following QC geometry optimization of randomly selected MD simulation snapshots using the quantum cluster model extended to include V293, P294 and E295 of cyt b. The energies are computed relative to the mean energy of snapshots with no electron transfer observed.

The $O_2/O_2^{\bullet-}$ binding pocket

The QC calculations in the present work are started from snapshots of MD simulations from an earlier study (Husen and Solov'yov, 2016) that identified a number of O_2 binding sites within the bc_1 complex by studying the diffusion of O_2 molecules initially added to the bulk water. Fig. S5a shows an O_2 molecule trapped in a binding pocket near the semiquinone ring and Fe_2S_2 after migrating from the membrane through the narrow channel occupied by the tail of $Q^{\bullet-}$ (Husen and Solov'yov, 2016)). Fig. S5b shows the distances of the bound O_2 molecule to nearby amino acids in the protein. The product state simulations in the present study are started from a snapshot with O_2 bound by changing charges and force-field parameters to convert O_2 to $O_2^{\bullet-}$ along corresponding changes to $Q^{\bullet-}$ or Fe_2S_2 depending on the model. Some small conformational changes of the binding pocket are observed, but $O_2^{\bullet-}$ remains trapped for a few nanoseconds, before it escapes into the water phase as illustrated in Fig. 6 (model I) and S6 (model II).

Superoxide unbinding in model II

A set of 100 molecular dynamics simulations of superoxide initially bound in the studied $O_2/O_2^{\bullet-}$ binding pocket near the Q_o-site of the bc_1 complex after electron transfer from the iron-sulfur cluster (model II) were carried out, similarly to the unbinding simulations for model I described in the main text. The results are shown in Fig. S6: Like for model I, $O_2^{\bullet-}$ exclusively escapes to the positive side of the membrane, i.e. the

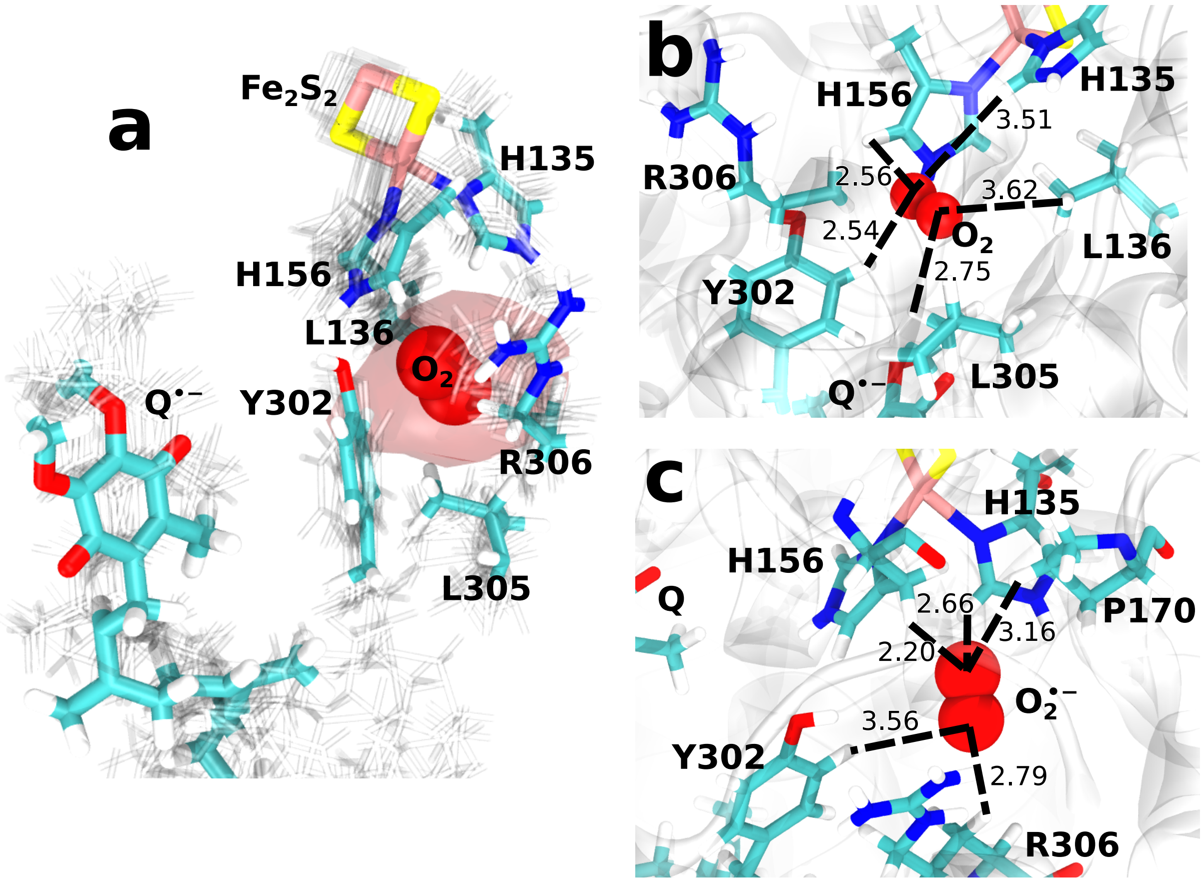

Figure S5. Binding pocket of O_2 and $O_2^{\bullet-}$ at the Qo-site (model I). A view of the O_2 binding pocket identified in an earlier study (Husen and Solov'yov, 2016), which also traps superoxide for a while after electron transfer. **a:** A snapshot of O_2 bound in the reactant state along with a transparent rendering of the protein and $Q^{\bullet-}$ in a small subset of other trajectory frames to illustrate the conformational variation (Husen and Solov'yov, 2016). The red surface indicates the localization of O_2 during a binding event. **b:** Close-up of the same snapshot with distances (in Å) between O_2 and nearby amino acids indicated. **c:** A snapshot from the product state simulations (model I) with $O_2^{\bullet-}$ bound in place of O_2 and $Q^{\bullet-}$ converted to Q.

intermembrane space in mitochondria, when it unbinds. The dynamics of $O_2^{\bullet-}$ on the way out appear to be more complex than for model I, but it generally unbinds a lot faster. Fig. S6b shows a characteristic binding time of 1.6 ns, when a threshold distance of 20 Å away from Fe_2S_2 is used to characterize unbinding.

Free energy perturbation simulations

Fig. S7 shows the results of the free energy perturbation (FEP) calculations for the two studied reactions. The results for the two sub-transformations (FEP1 and FEP2) in each model have been combined, such that the rightmost value in the diagram indicates the total free energy change, ΔG^0, for the studied reaction.

In FEP calculations, the O_2 molecule was kept within a small region with a radius of 8 Å and 10 Å for model I and II, respectively, by means of an artificial confining potential to prevent it from leaving the position near Fe_2S_2 entirely. For model I, the center of the confined region was chosen as the midpoint between the carbonyl carbon atoms of TYR302 of cytochrome b and HIS135 of the ISP, and for model II, the midpoint between the closest oxygen atom of $Q^{\bullet-}$ and iron atom of Fe_2S_2 was used. The confining potential is constantly zero within the boundary and takes the shape of a half-harmonic potential on the outside. In other words, the O_2 molecule only feels the confining potential, if it pushes against the boundary.

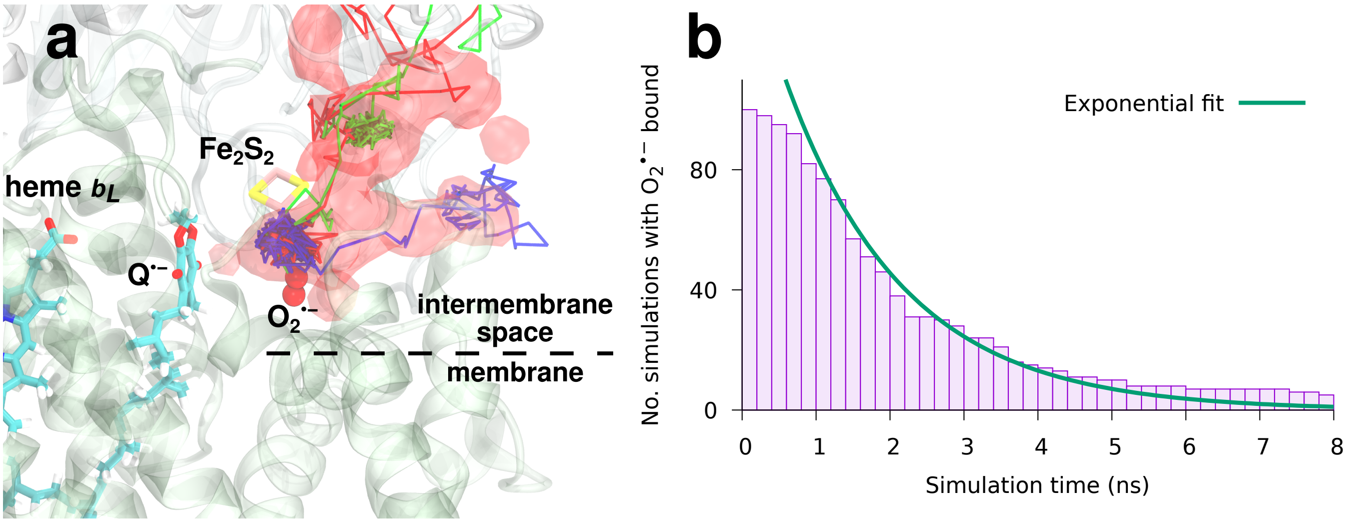

Figure S6. The dynamics and binding of a newly formed superoxide (model II). $O_2^{\bullet-}$ stays bound for a while in the same binding pocket as O_2 near the Q_o-site, but eventually escapes into the bulk water. **a:** Superoxide escapes into the intermembrane space. The red regions indicate the averaged localization of O_2 during all 100 simulated unbinding events. Three example trajectories are shown with red, green and blue colors. **b:** A histogram of the number $N_b(t)$ of simulations that still has $O_2^{\bullet-}$ bound after a specified simulation time along with a fit of an exponential model, Eq. (6), with a characteristic binding time of $\tau = 1.6\,\mathrm{ns}$.

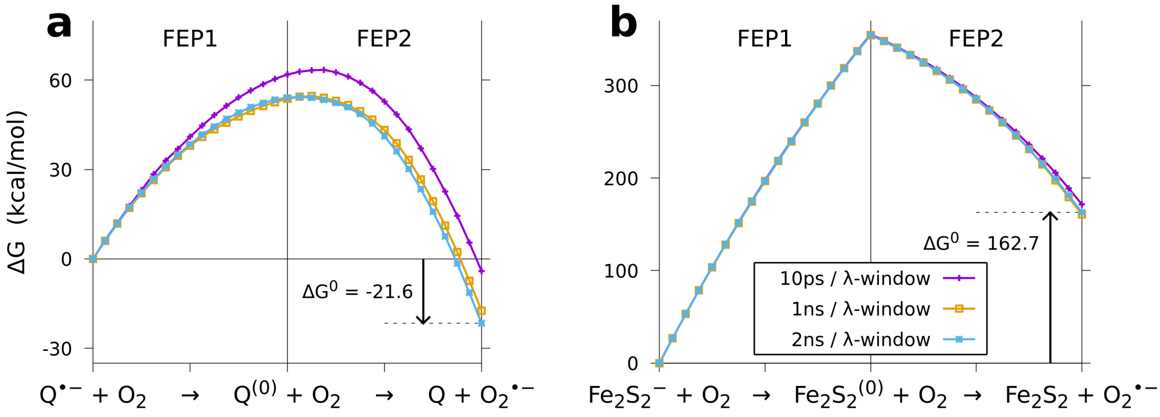

Figure S7. Combined free energy change from reactant to product state. The figure shows the combined results of two partial FEP transformations, FEP1 and FEP2, taking the system from the reactant state, through the intermediate neutral state and finally to the product state charge distribution. The free energy change ΔG is computed relative to the reactant state, so the rightmost value in the diagram is the total free energy change ΔG^0, corresponding to a conversion of the reactant into the product. The values of ΔG^0 indicated by arrows are in kcal/mol and are taken from the calculations with 2 ns simulation time per λ-window, i.e. the blue curves in the diagram. **a:** results for model I, **b:** results for model II.

If the O_2 molecule remains naturally within the bounded region most of the time, the effect of the artificial potential on the measured free energy of the system should be minimal.

Fig. S8 shows the distribution of the bounded distance keeping O_2 at the binding site. For most simulations, the O_2 molecule remains naturally within the bounded region, and the constraint only serves to prevent rare events of O_2 leaving the binding site. In this case, we assume the free energy penalty due to the constraint as negligible. In the FEP2 transformation for model I, however, the O_2 molecule spends a significant

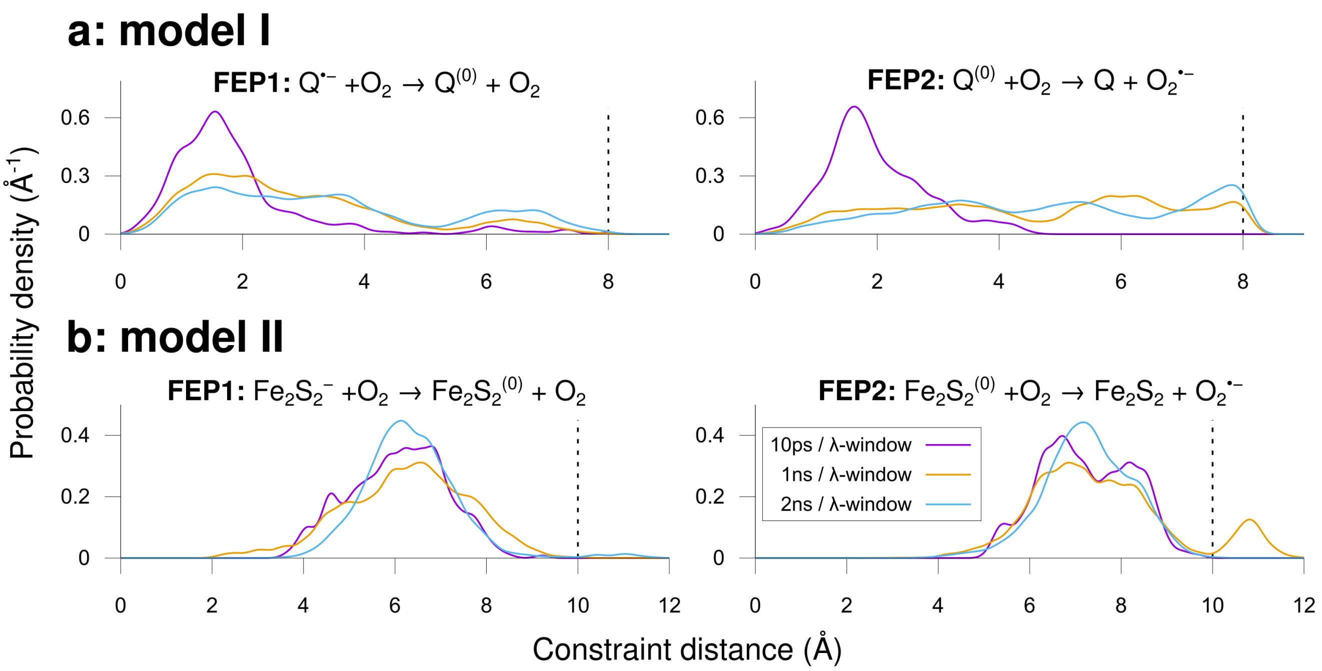

Figure S8. Distribution of the constrained distance preventing O_2 from leaving the bound position near the Q_o-site. In model I (**a**), the constrained distance is between O_2 and the midpoint between TYR302 of cytochrome b and HIS135 of the ISP, and the upper distance limit is 8 Å, indicated by a dashed line. For model II (**b**), the constrained distance is defined as the distance between O_2 and the midpoint between the Fe2 iron atom (see Fig. 3) of Fe_2S_2 and the closest oxygen atom of $Q^{\bullet-}$, and the upper distance limit is 10 Å. The colors indicate different durations of the λ-windows used in the simulations.

amount of time near edge of the boundary, which could lead to an error in the free energy calculation. Fig. S9 shows the mean and standard deviation of the constraint distance at each λ-window of the FEP2 transformation for model I. During the backward simulations, the O_2 molecule appears to jump back and forth between a local minimum near its initial position and an alternative position near the boundary of the confined region. However, the O_2 molecule primarily pushes against the boundary at the unphysical intermediate stages of the FEP transition and less so at the final fully coupled product state, indicating that the constraint should not significantly disturb the estimated end-to-end free energy difference between the reactant and the product states.

Raw free energy perturbation results

In this section, the results from the FEP calculations are presented in more detail. Figs. S10–S21 shows the free energy results from the forward and backward transformations as well as the distribution of the sampled potential energy difference ΔU and the convergence of the estimated free energy change for each λ-window. The 12 figures cover the two models, the two partial FEP tranformations (FEP1 and FEP2) for each model and three choices of the simulation length per λ-window (see Fig. S1 in the main text). Tables S3 and S4 summarize the results. The sum $\Delta G_{\text{forward}} + \Delta G_{\text{backward}}$ indicates the discrepancy between results from the forward and the backward transformations: if perfect micro-reversibility is achieved, the sum should be zero Dixit and Chipot (2001). ΔG_{BAR} is calculated from a combination of the forward and backward data using the Bennet acceptance-ratio (BAR) estimator Bennett (1976). The ParseFEP plugin

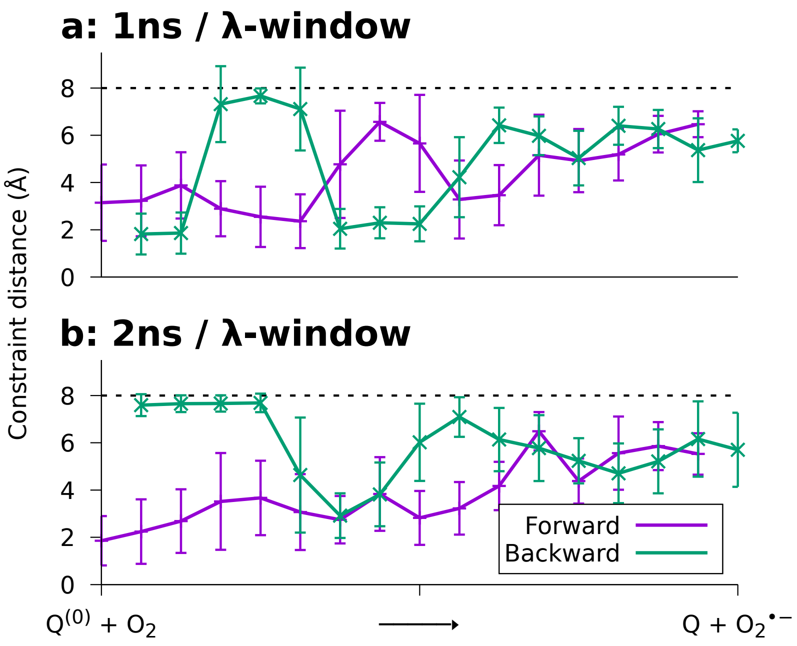

Figure S9. Time evolution of the constrained distance for the FEP2 transition in model I. The curves and error bars indicate the means and standard deviations of the constrained distance between O_2 and a reference point at the Q_o-site in each of the 16 λ windows of the FEP2 simulations going from the neutral to the product state in model I. Data for both the forward and backward transitions and for two values of the λ-window length is shown. The threshold distance of 8 Å between O_2 and the center of the confined region is indicated with a dashed line.

Liu et al. (2012) of VMD Humphrey et al. (1996) was used for data analysis, including calculation of the BAR estimator, and generation of the figures.

Model I: $Q^{\bullet -} + O_2 \rightarrow Q + O_2^{\bullet -}$

	Simulation time per λ-window	10 ps	1 ns	2 ns
FEP1	$\Delta G_{\text{forward}}$	65.76	54.96	56.00
	$\Delta G_{\text{forward}} + \Delta G_{\text{backward}}$	7.88	2.07	3.50
	ΔG_{BAR}	61.87	53.72	54.14
FEP2	$\Delta G_{\text{forward}}$	-58.74	-70.13	-73.46
	$\Delta G_{\text{forward}} + \Delta G_{\text{backward}}$	14.10	2.60	5.05
	ΔG_{BAR}	-65.96	-71.13	-75.78
Combined	$\Delta G^0 = \Delta G_{\text{BAR,FEP1}} + \Delta G_{\text{BAR,FEP2}}$	-4.09	-17.41	-21.64

Table S3. Results of free energy perturbation calculations for model I. The free energy change during the forward transformation, $\Delta G_{\text{forward}}$, the forward/backward discrepancy, $\Delta G_{\text{forward}} + \Delta G_{\text{backward}}$, as well as estimates based on combined data from the forward and backward transformations using the BAR estimator, ΔG_{BAR}, are shown in the table separately for the FEP1 and FEP2 transformations and for three different settings of the simulation length. ΔG^0 is the combined estimate of the reactant–product free energy change. All energies are in kcal/mol.

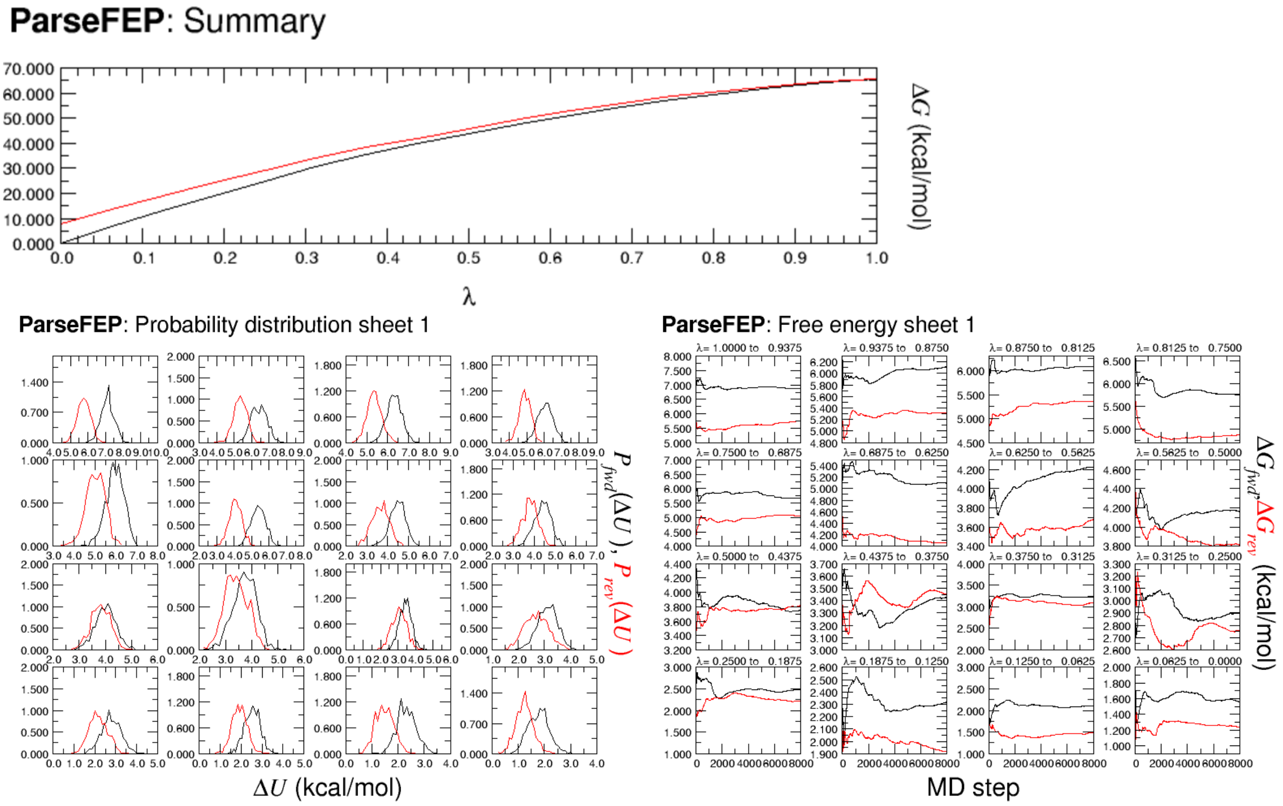

Figure S10. Results of the FEP1 calculations for model I with 10 ps/λ-window. *Top:* the accumulated free energy change ΔG is shown as a function of λ in the forward (black curve) and backward (red curve) transformations, respectively. The two curves have been connected at $\lambda = 1$, such that the value of the red curve at $\lambda = 0$ indicates the discrepancy between the results from the forward and backward transformations (see Table S3). *Bottom left:* the sampled probability distributions, $P_{frwd}(\Delta U)$ and $P_{rev}(\Delta U)$, of the potential energy change ΔU for going from reactant to product state at a given set of atomic coordinates is shown for each λ window for the forward (black) and backward (red) transformations, respectively. *Bottom right:* the convergence of the estimated free energy change within each simulated λ-window in the forward, ΔG_{frwd}, and backward, ΔG_{rev}, transformations.

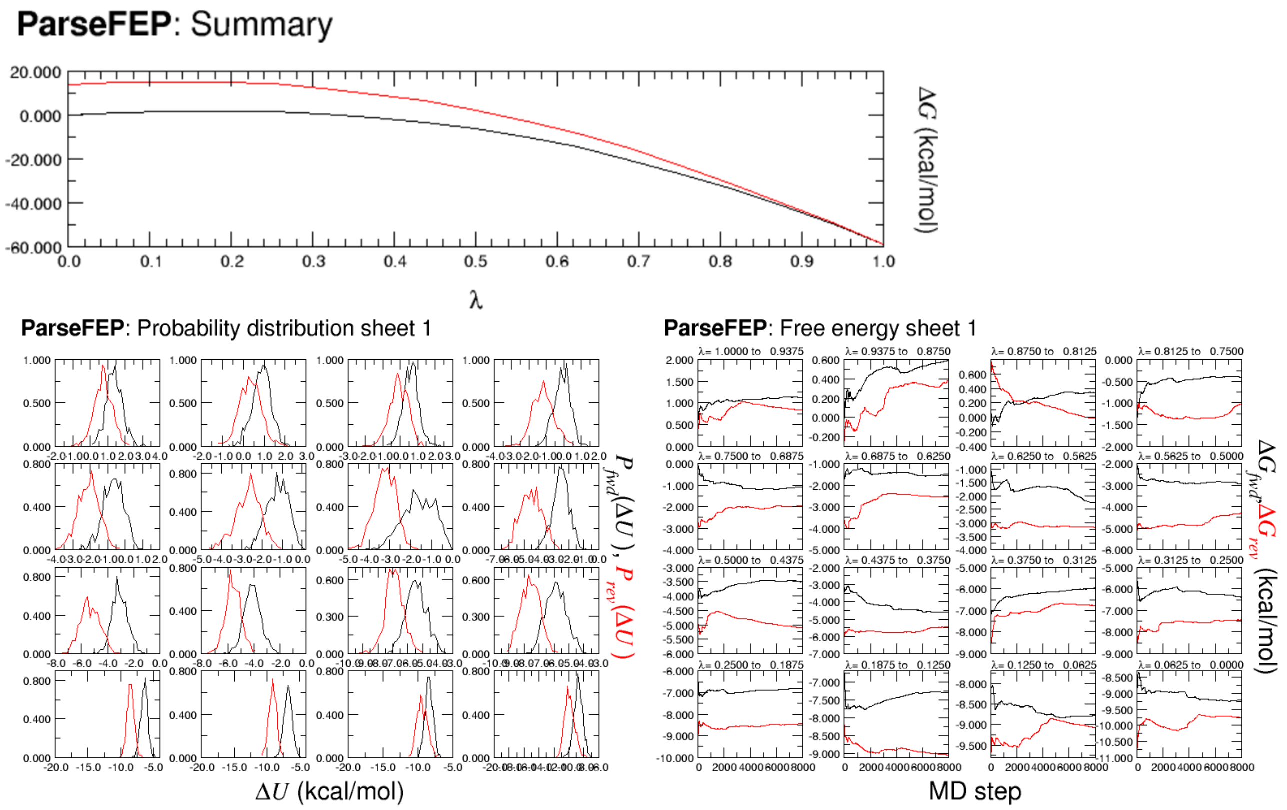

Figure S11. Results of the FEP2 calculations for model I with 10 ps/λ-window. *Top:* the accumulated free energy change ΔG is shown as a function of λ in the forward (black curve) and backward (red curve) transformations, respectively. The two curves have been connected at $\lambda = 1$, such that the value of the red curve at $\lambda = 0$ indicates the discrepancy between the results from the forward and backward transformations (see Table S3). *Bottom left:* the sampled probability distributions, $P_{frwd}(\Delta U)$ and $P_{rev}(\Delta U)$, of the potential energy change ΔU for going from reactant to product state at a given set of atomic coordinates is shown for each λ window for the forward (black) and backward (red) transformations, respectively. *Bottom right:* the convergence of the estimated free energy change within each simulated λ-window in the forward, ΔG_{frwd}, and backward, ΔG_{rev}, transformations.

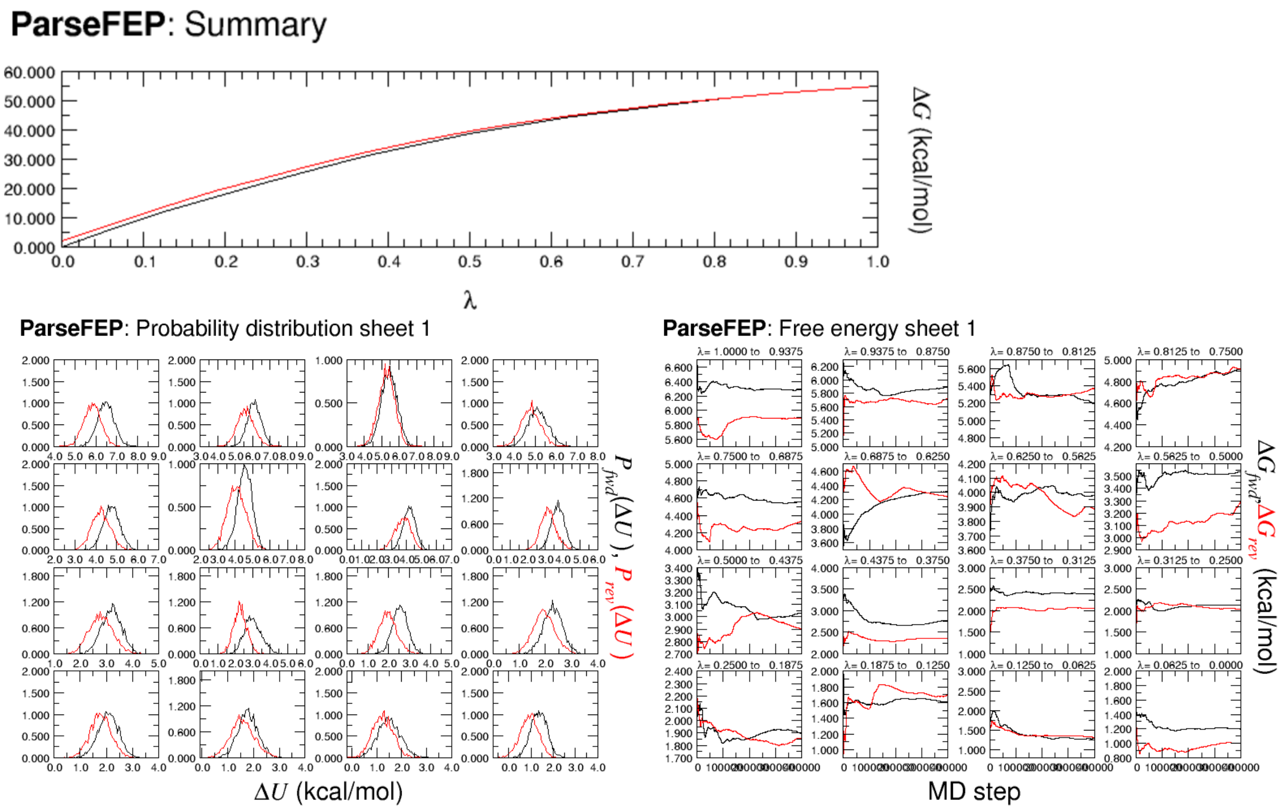

Figure S12. Results of the FEP1 calculations for model I with 1 ns/λ-window. *Top:* the accumulated free energy change ΔG is shown as a function of λ in the forward (black curve) and backward (red curve) transformations, respectively. The two curves have been connected at $\lambda = 1$, such that the value of the red curve at $\lambda = 0$ indicates the discrepancy between the results from the forward and backward transformations (see Table S3). *Bottom left:* the sampled probability distributions, $P_{frwd}(\Delta U)$ and $P_{rev}(\Delta U)$, of the potential energy change ΔU for going from reactant to product state at a given set of atomic coordinates is shown for each λ window for the forward (black) and backward (red) transformations, respectively. *Bottom right:* the convergence of the estimated free energy change within each simulated λ-window in the forward, ΔG_{frwd}, and backward, ΔG_{rev}, transformations.

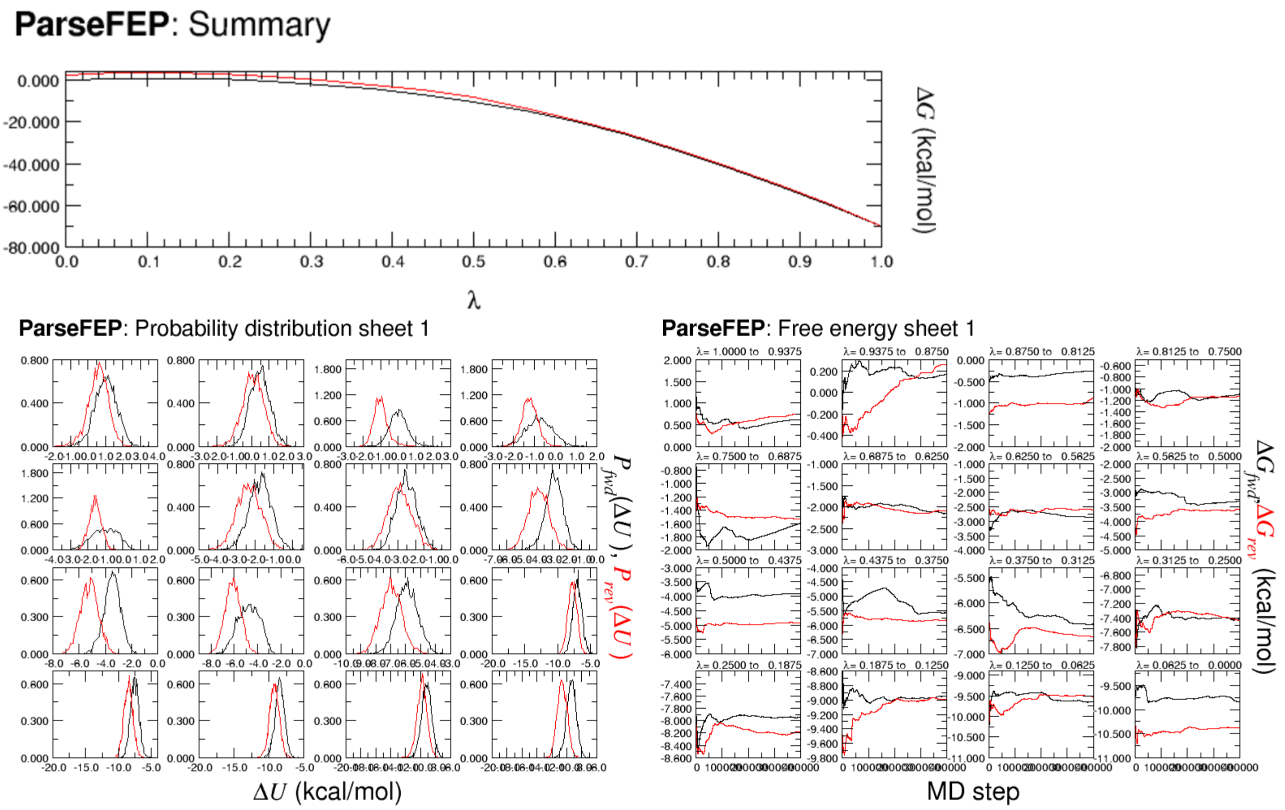

Figure S13. Results of the FEP2 calculations for model I with 1 ns/λ-window. *Top:* the accumulated free energy change ΔG is shown as a function of λ in the forward (black curve) and backward (red curve) transformations, respectively. The two curves have been connected at $\lambda = 1$, such that the value of the red curve at $\lambda = 0$ indicates the discrepancy between the results from the forward and backward transformations (see Table S3). *Bottom left:* the sampled probability distributions, $P_{frwd}(\Delta U)$ and $P_{rev}(\Delta U)$, of the potential energy change ΔU for going from reactant to product state at a given set of atomic coordinates is shown for each λ window for the forward (black) and backward (red) transformations, respectively. *Bottom right:* the convergence of the estimated free energy change within each simulated λ-window in the forward, ΔG_{frwd}, and backward, ΔG_{rev}, transformations.

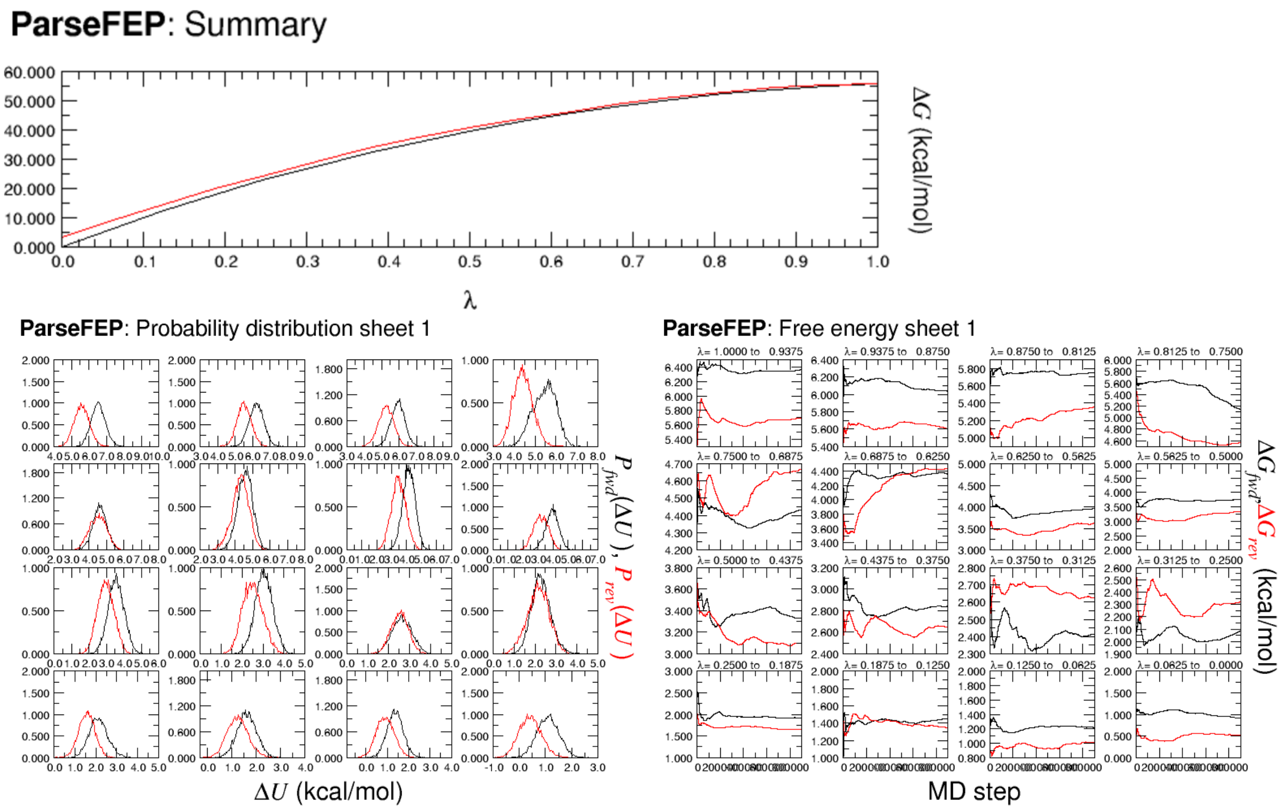

Figure S14. Results of the FEP1 calculations for model I with 2 ns/λ-window. *Top:* the accumulated free energy change ΔG is shown as a function of λ in the forward (black curve) and backward (red curve) transformations, respectively. The two curves have been connected at $\lambda = 1$, such that the value of the red curve at $\lambda = 0$ indicates the discrepancy between the results from the forward and backward transformations (see Table S3). *Bottom left:* the sampled probability distributions, $P_{frwd}(\Delta U)$ and $P_{rev}(\Delta U)$, of the potential energy change ΔU for going from reactant to product state at a given set of atomic coordinates is shown for each λ window for the forward (black) and backward (red) transformations, respectively. *Bottom right:* the convergence of the estimated free energy change within each simulated λ-window in the forward, ΔG_{frwd}, and backward, ΔG_{rev}, transformations.

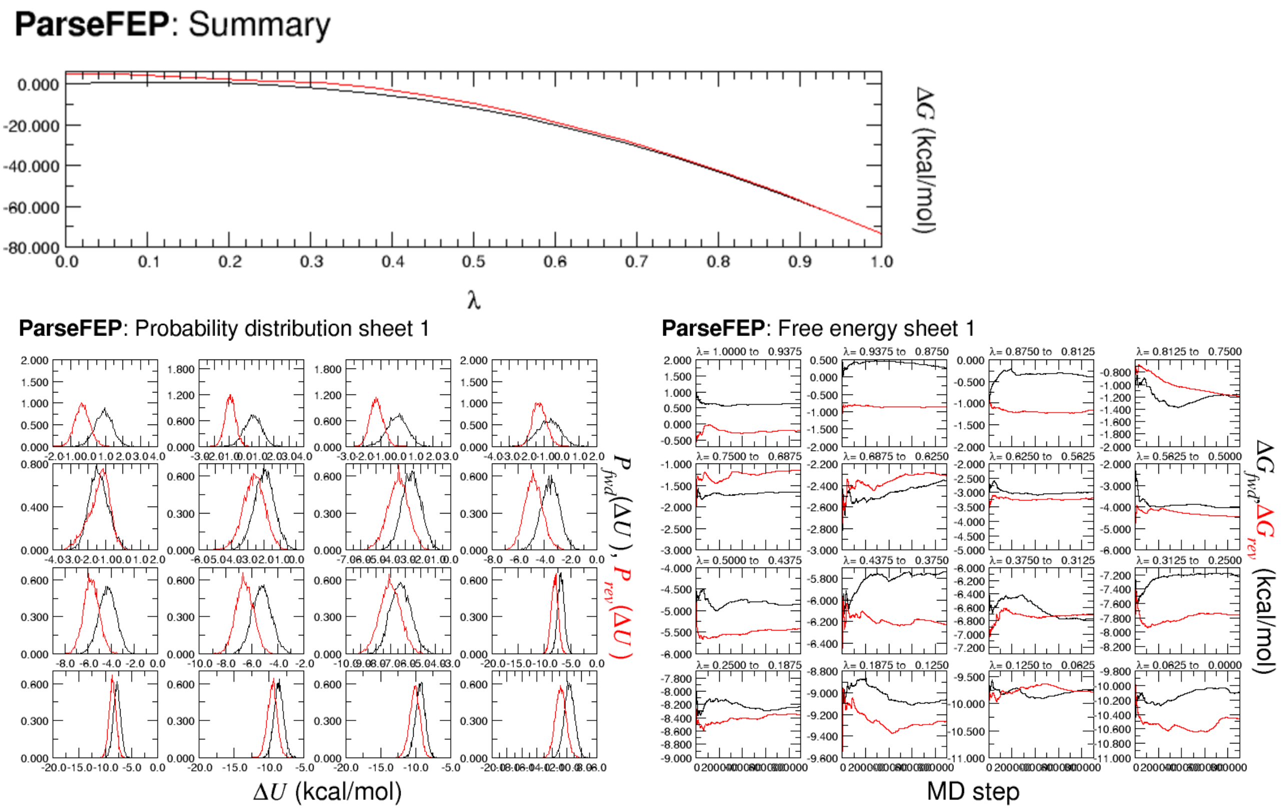

Figure S15. Results of the FEP2 calculations for model I with 2 ns/λ-window. *Top:* the accumulated free energy change ΔG is shown as a function of λ in the forward (black curve) and backward (red curve) transformations, respectively. The two curves have been connected at $\lambda = 1$, such that the value of the red curve at $\lambda = 0$ indicates the discrepancy between the results from the forward and backward transformations (see Table S3). *Bottom left:* the sampled probability distributions, $P_{frwd}(\Delta U)$ and $P_{rev}(\Delta U)$, of the potential energy change ΔU for going from reactant to product state at a given set of atomic coordinates is shown for each λ window for the forward (black) and backward (red) transformations, respectively. *Bottom right:* the convergence of the estimated free energy change within each simulated λ-window in the forward, ΔG_{frwd}, and backward, ΔG_{rev}, transformations.

Model II: $Fe_2S_2^- + O_2 \rightarrow Fe_2S_2 + O_2^{\bullet -}$

	Simulation time per λ-window	10 ps	1 ns	2 ns
FEP1	$\Delta G_{\text{forward}}$	357.76	355.68	354.83
	$\Delta G_{\text{forward}} + \Delta G_{\text{backward}}$	5.09	2.58	1.24
	ΔG_{BAR}	355.12	354.27	354.24
FEP2	$\Delta G_{\text{forward}}$	-177.38	-191.27	-191.00
	$\Delta G_{\text{forward}} + \Delta G_{\text{backward}}$	12.45	4.51	1.01
	ΔG_{BAR}	-183.63	-193.68	-191.53
Combined	$\Delta G^0 = \Delta G_{\text{BAR,FEP1}} + \Delta G_{\text{BAR,FEP2}}$	171.49	160.58	162.72

Table S4. Results of free energy perturbation calculations for model II. The free energy change during the forward transformation, $\Delta G_{\text{forward}}$, the forward/backward discrepancy, $\Delta G_{\text{forward}} + \Delta G_{\text{backward}}$, as well as estimates based on combined data from the forward and backward transformations using the BAR estimator, ΔG_{BAR}, are shown in the table separately for the FEP1 and FEP2 transformations and for three different settings of the simulation length. ΔG^0 is the combined estimate of the reactant–product free energy change. All energies are in kcal/mol.

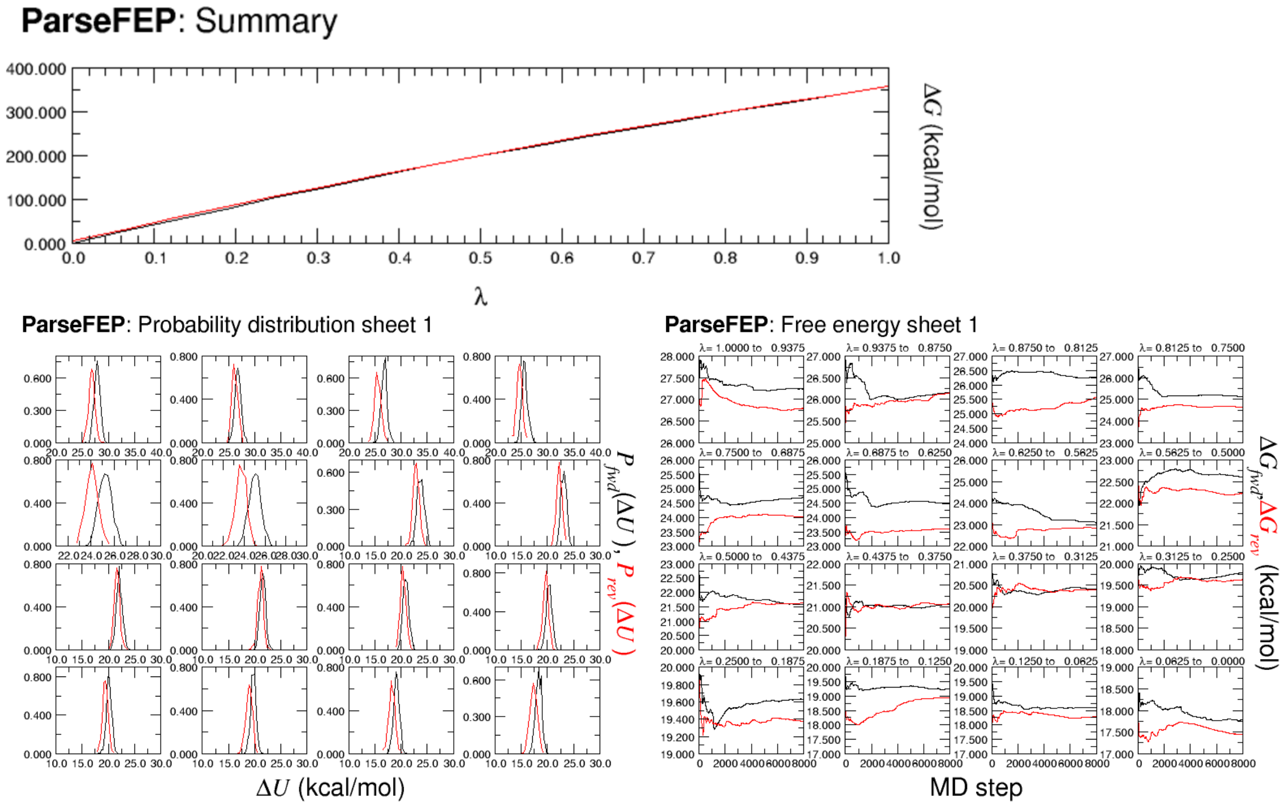

Figure S16. Results of the FEP1 calculations for model II with 10 ps/λ-window. *Top:* the accumulated free energy change ΔG is shown as a function of λ in the forward (black curve) and backward (red curve) transformations, respectively. The two curves have been connected at $\lambda = 1$, such that the value of the red curve at $\lambda = 0$ indicates the discrepancy between the results from the forward and backward transformations (see Table S4). *Bottom left:* the sampled probability distributions, $P_{frwd}(\Delta U)$ and $P_{rev}(\Delta U)$, of the potential energy change ΔU for going from reactant to product state at a given set of atomic coordinates is shown for each λ window for the forward (black) and backward (red) transformations, respectively. *Bottom right:* the convergence of the estimated free energy change within each simulated λ-window in the forward, ΔG_{frwd}, and backward, ΔG_{rev}, transformations.

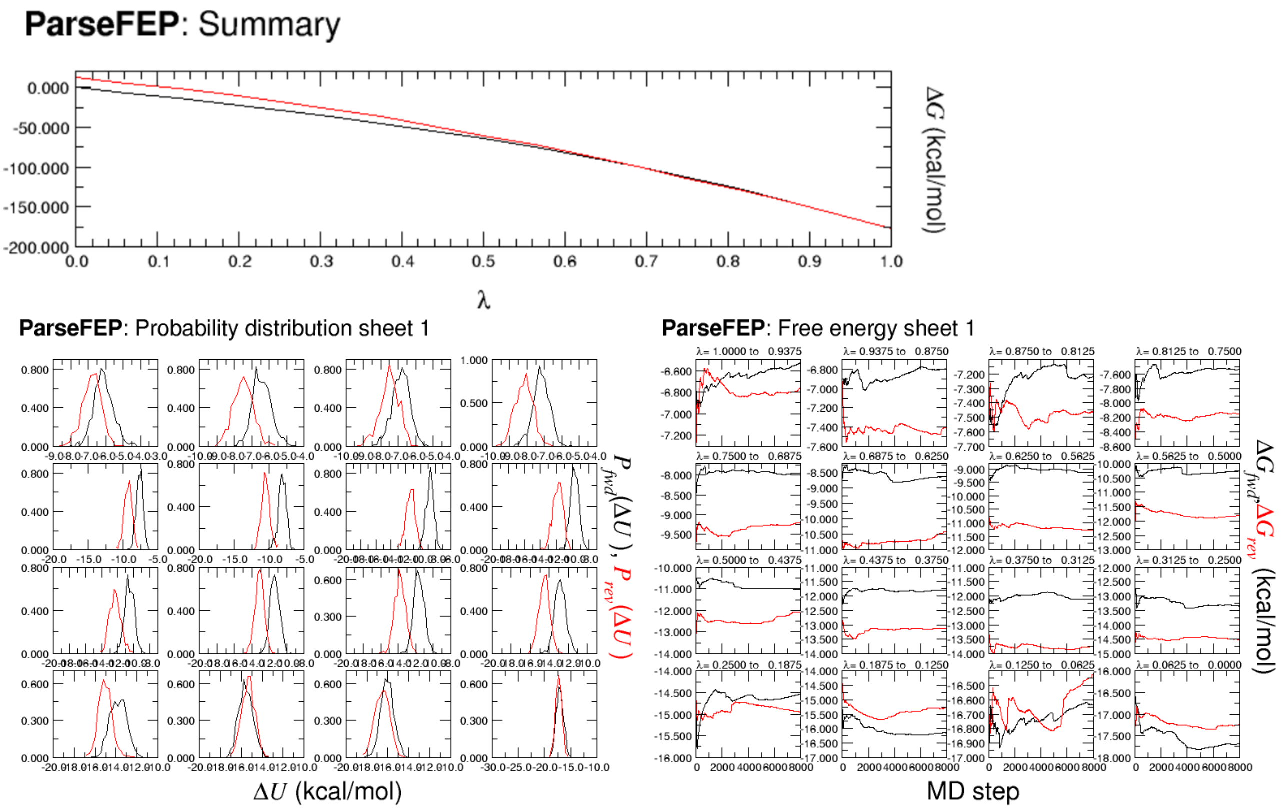

Figure S17. Results of the FEP2 calculations for model II with 10 ps/λ-window. *Top:* the accumulated free energy change ΔG is shown as a function of λ in the forward (black curve) and backward (red curve) transformations, respectively. The two curves have been connected at $\lambda = 1$, such that the value of the red curve at $\lambda = 0$ indicates the discrepancy between the results from the forward and backward transformations (see Table S4). *Bottom left:* the sampled probability distributions, $P_{frwd}(\Delta U)$ and $P_{rev}(\Delta U)$, of the potential energy change ΔU for going from reactant to product state at a given set of atomic coordinates is shown for each λ window for the forward (black) and backward (red) transformations, respectively. *Bottom right:* the convergence of the estimated free energy change within each simulated λ-window in the forward, ΔG_{frwd}, and backward, ΔG_{rev}, transformations.

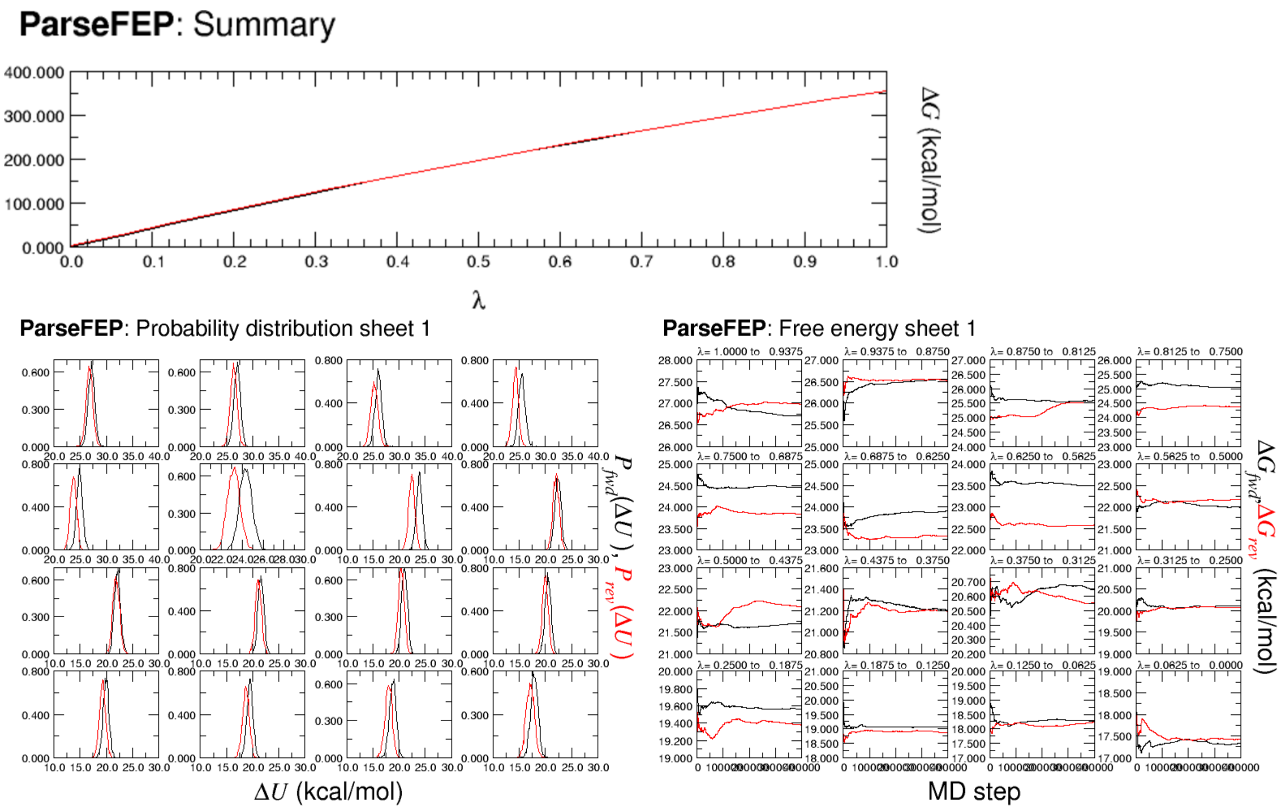

Figure S18. Results of the FEP1 calculations for model II with 1 ns/λ-window. *Top:* the accumulated free energy change ΔG is shown as a function of λ in the forward (black curve) and backward (red curve) transformations, respectively. The two curves have been connected at $\lambda = 1$, such that the value of the red curve at $\lambda = 0$ indicates the discrepancy between the results from the forward and backward transformations (see Table S4). *Bottom left:* the sampled probability distributions, $P_{frwd}(\Delta U)$ and $P_{rev}(\Delta U)$, of the potential energy change ΔU for going from reactant to product state at a given set of atomic coordinates is shown for each λ window for the forward (black) and backward (red) transformations, respectively. *Bottom right:* the convergence of the estimated free energy change within each simulated λ-window in the forward, ΔG_{frwd}, and backward, ΔG_{rev}, transformations.

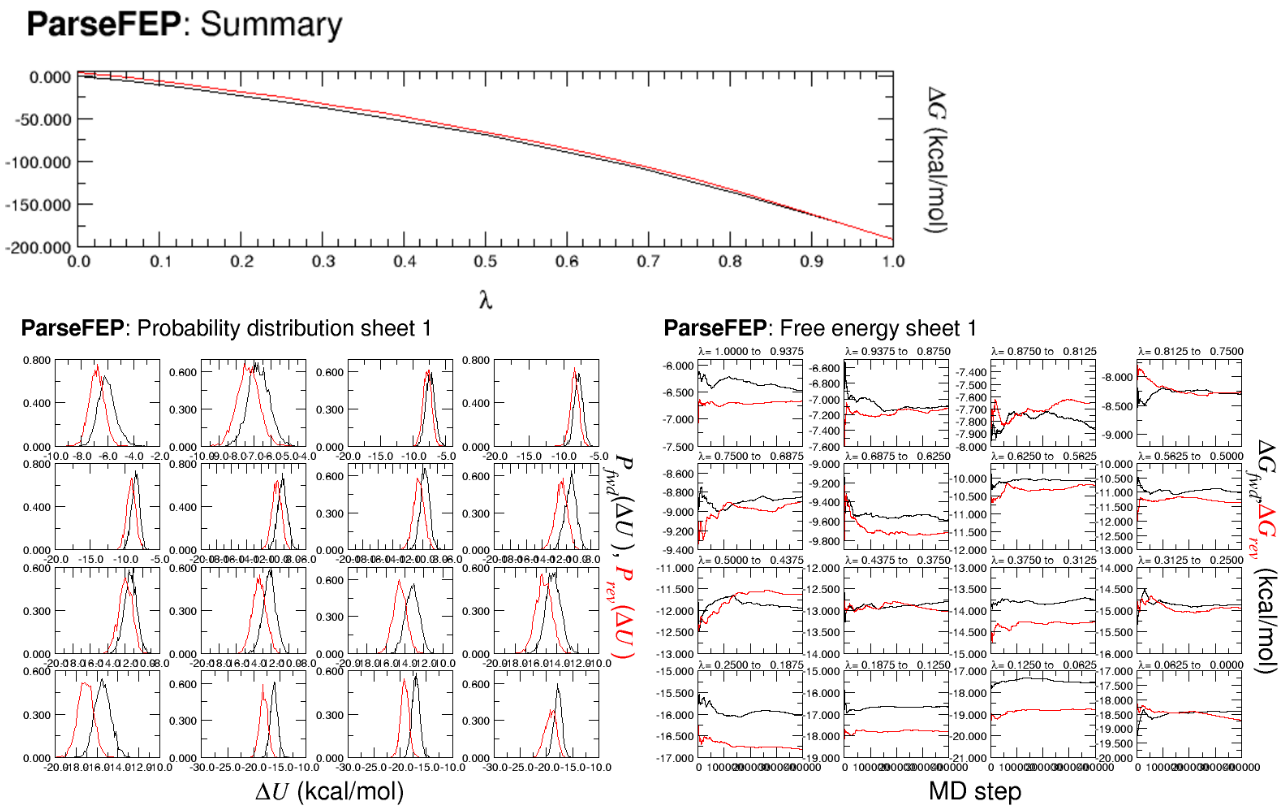

Figure S19. Results of the FEP2 calculations for model II with 1 ns/λ-window. *Top:* the accumulated free energy change ΔG is shown as a function of λ in the forward (black curve) and backward (red curve) transformations, respectively. The two curves have been connected at $\lambda = 1$, such that the value of the red curve at $\lambda = 0$ indicates the discrepancy between the results from the forward and backward transformations (see Table S4). *Bottom left:* the sampled probability distributions, $P_{frwd}(\Delta U)$ and $P_{rev}(\Delta U)$, of the potential energy change ΔU for going from reactant to product state at a given set of atomic coordinates is shown for each λ window for the forward (black) and backward (red) transformations, respectively. *Bottom right:* the convergence of the estimated free energy change within each simulated λ-window in the forward, ΔG_{frwd}, and backward, ΔG_{rev}, transformations.

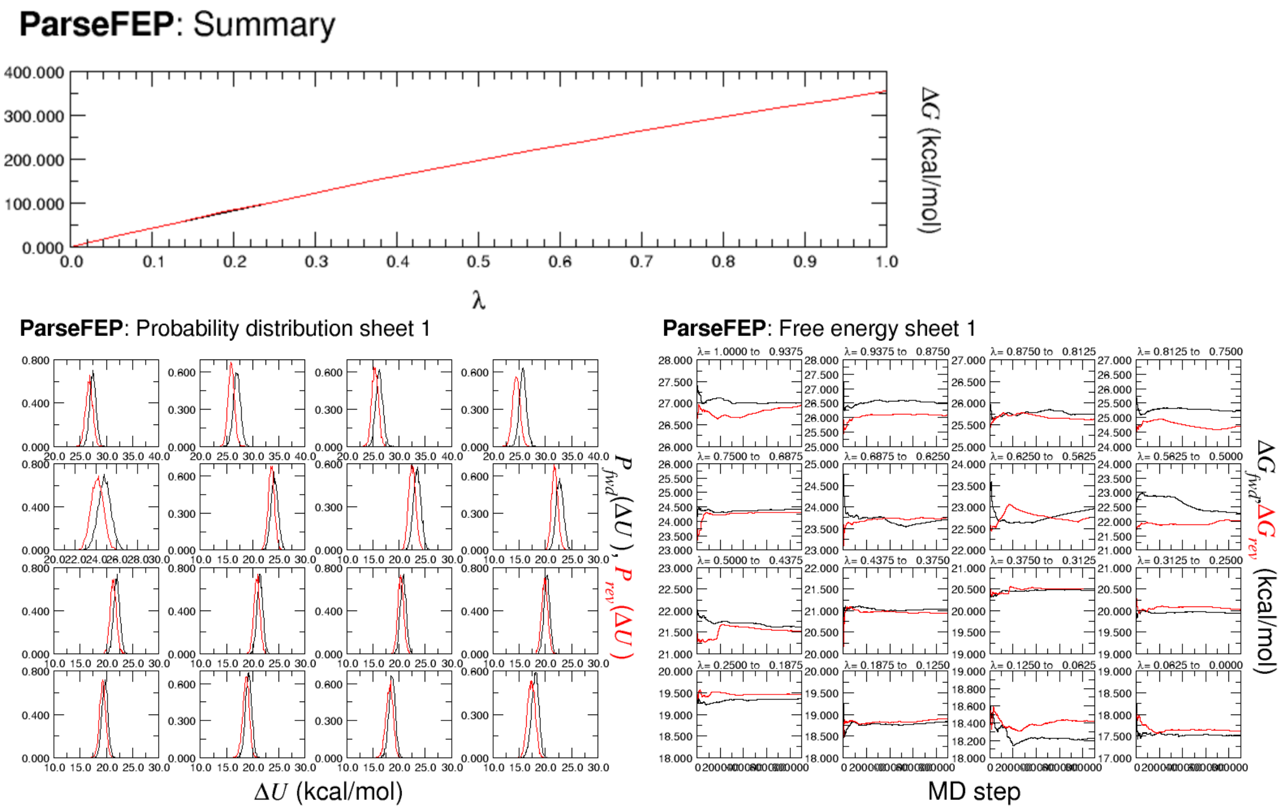

Figure S20. Results of the FEP1 calculations for model II with 2 ns/λ-window. *Top:* the accumulated free energy change ΔG is shown as a function of λ in the forward (black curve) and backward (red curve) transformations, respectively. The two curves have been connected at $\lambda = 1$, such that the value of the red curve at $\lambda = 0$ indicates the discrepancy between the results from the forward and backward transformations (see Table S4). *Bottom left:* the sampled probability distributions, $P_{frwd}(\Delta U)$ and $P_{rev}(\Delta U)$, of the potential energy change ΔU for going from reactant to product state at a given set of atomic coordinates is shown for each λ window for the forward (black) and backward (red) transformations, respectively. *Bottom right:* the convergence of the estimated free energy change within each simulated λ-window in the forward, ΔG_{frwd}, and backward, ΔG_{rev}, transformations.

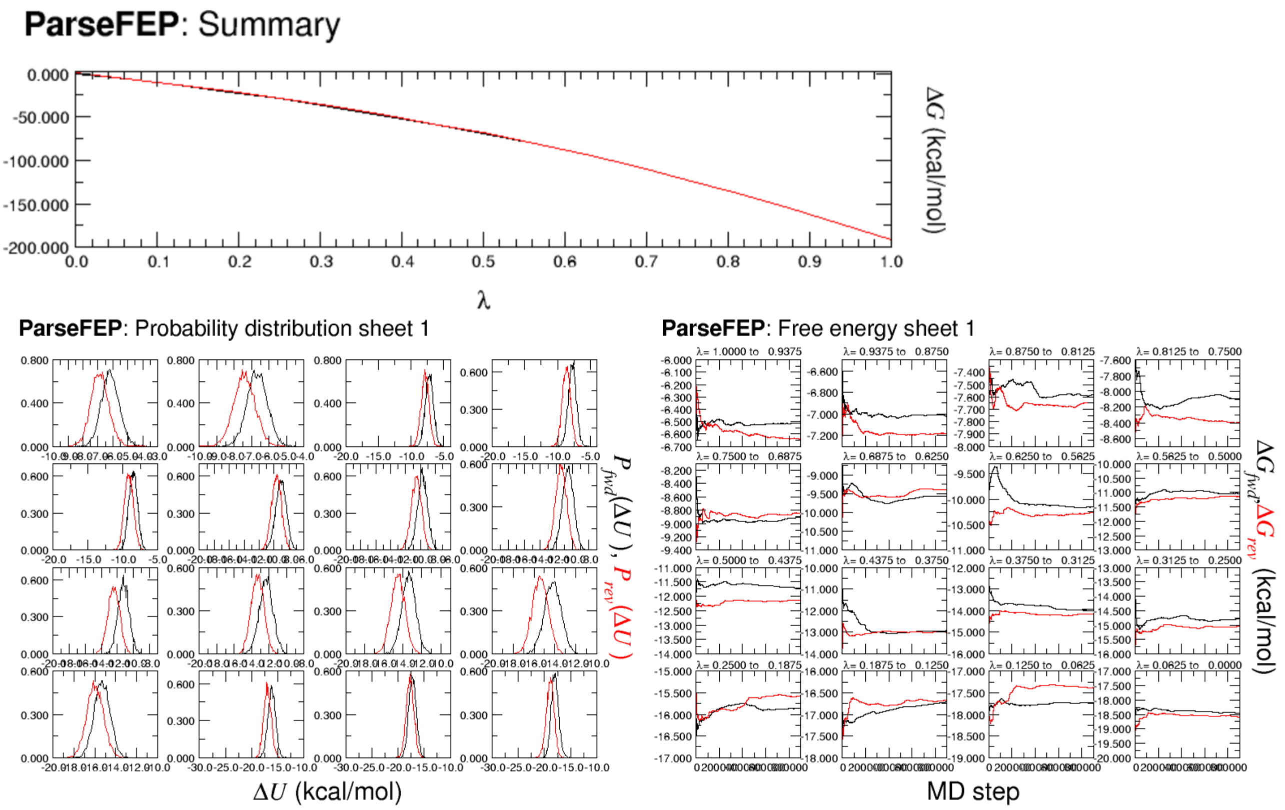

Figure S21. Results of the FEP2 calculations for model II with 2 ns/λ-window. *Top:* the accumulated free energy change ΔG is shown as a function of λ in the forward (black curve) and backward (red curve) transformations, respectively. The two curves have been connected at $\lambda = 1$, such that the value of the red curve at $\lambda = 0$ indicates the discrepancy between the results from the forward and backward transformations (see Table S4). *Bottom left:* the sampled probability distributions, $P_{frwd}(\Delta U)$ and $P_{rev}(\Delta U)$, of the potential energy change ΔU for going from reactant to product state at a given set of atomic coordinates is shown for each λ window for the forward (black) and backward (red) transformations, respectively. *Bottom right:* the convergence of the estimated free energy change within each simulated λ-window in the forward, ΔG_{frwd}, and backward, ΔG_{rev}, transformations.

REFERENCES

Barragan, A. M., Crofts, A. R., Schulten, K., and Solov'yov, I. A. (2015). Identification of ubiquinol binding motifs at the Q_o-site of the cytochrome bc_1 complex. *J. Phys. Chem. B* 119, 433–447

Barragan, A. M., Schulten, K., and Solov'yov, I. A. (2016). Mechanism of the primary charge transfer reaction in the cytochrome bc_1 complex. *J. Phys. Chem. B* 120, 11369–11380

Bennett, C. H. (1976). Efficient estimation of free energy differences from monte carlo data. *J. Comp. Phys.* 22, 245–268

Crofts, A. R., Barquera, B., Gennis, R. B., Kuras, R., Guergova-Kuras, M., and Berry, E. A. (1999). Mechanism of ubiquinol oxidation by the bc_1 complex: different domains of the quinol binding pocket and their role in the mechanism and binding of inhibitors. *Biochemistry* 38, 15807–15826

Dixit, S. B. and Chipot, C. (2001). Can absolute free energies of association be estimated from molecular mechanical simulations? The biotin- streptavidin system revisited. *J. Phys. Chem. A* 105, 9795–9799

Humphrey, W., Dalke, A., and Schulten, K. (1996). VMD – Visual Molecular Dynamics. *J. Mol. Graph.* 14, 33–38

Husen, P. and Solov'yov, I. A. (2016). Spontaneous binding of molecular oxygen at the Q_o-site of the bc_1 complex could stimulate superoxide formation. *J. Am. Chem. Soc.* 138, 12150–12158

Liu, P., Dehez, F., Cai, W., and Chipot, C. (2012). A toolkit for the analysis of free-energy perturbation calculations. *J. Chem. Theory Comput.* 8, 2606–2616

Postila, P., Kaszuba, K., Sarewicz, M., Osyczka, A., Vattulainen, I., and Róg, T. (2013). Key role of water in proton transfer at the Q_o-site of the cytochrome bc_1 complex predicted by atomistic molecular dynamics simulations. *Biochim. Biophys. Acta* 1827, 761–768
